# Recently photoassimilated carbon and fungus‐delivered nitrogen are spatially correlated in the ectomycorrhizal tissue of *Fagus sylvatica*

**DOI:** 10.1111/nph.17591

**Published:** 2021-08-06

**Authors:** Werner Mayerhofer, Arno Schintlmeister, Marlies Dietrich, Stefan Gorka, Julia Wiesenbauer, Victoria Martin, Raphael Gabriel, Siegfried Reipert, Marieluise Weidinger, Peta Clode, Michael Wagner, Dagmar Woebken, Andreas Richter, Christina Kaiser

**Affiliations:** ^1^ Centre for Microbiology and Environmental Systems Science University of Vienna Vienna A‐1030 Austria; ^2^ Large‐Instrument Facility for Environmental and Isotope Mass Spectrometry University of Vienna Vienna A‐1030 Austria; ^3^ Core Facility Cell Imaging and Ultrastructure Research University of Vienna Vienna A‐1030 Austria; ^4^ Centre for Microscopy, Characterisation & Analysis University of Western Australia Perth WA 6009 Australia; ^5^ Department of Chemistry and Bioscience Aalborg University Aalborg DK‐9220 Denmark

**Keywords:** carbon, ectomycorrhiza, *Fagus sylvatica* (beech), NanoSIMS, nitrogen (N), recent photosynthates, reciprocal rewards, resource exchange

## Abstract

- Ectomycorrhizal plants trade plant‐assimilated carbon for soil nutrients with their fungal partners. The underlying mechanisms, however, are not fully understood. Here we investigate the exchange of carbon for nitrogen in the ectomycorrhizal symbiosis of *Fagus sylvatica* across different spatial scales from the root system to the cellular level.
- We provided ^15^N‐labelled nitrogen to mycorrhizal hyphae associated with one half of the root system of young beech trees, while exposing plants to a ^13^CO_2_ atmosphere. We analysed the short‐term distribution of ^13^C and ^15^N in the root system with isotope‐ratio mass spectrometry, and at the cellular scale within a mycorrhizal root tip with nanoscale secondary ion mass spectrometry (NanoSIMS).
- At the root system scale, plants did not allocate more ^13^C to root parts that received more ^15^N. Nanoscale secondary ion mass spectrometry imaging, however, revealed a highly heterogenous, and spatially significantly correlated distribution of ^13^C and ^15^N at the cellular scale.
- Our results indicate that, on a coarse scale, plants do not allocate a larger proportion of photoassimilated C to root parts associated with N‐delivering ectomycorrhizal fungi. Within the ectomycorrhizal tissue, however, recently plant‐assimilated C and fungus‐delivered N were spatially strongly coupled. Here, NanoSIMS visualisation provides an initial insight into the regulation of ectomycorrhizal C and N exchange at the microscale.

## Introduction

Ectomycorrhizal (EM) fungi play an important role in the nitrogen (N) nutrition of trees in boreal and temperate forests. They use their extensive extraradical hyphal network to take up organic and inorganic N compounds from the forest soil, and deliver N to their host plants in exchange for carbon (C). Moreover, ectomycorrhizal fungi are able to facilitate the decomposition of complex soil organic N by excreting extracellular enzymes (Shah *et al*., 2013; Lindahl & Tunlid, 2015; Wang *et al*., 2020), employing oxidative mechanisms (Shah *et al*., 2016) or stimulating free‐living microbial decomposers (Gorka *et al*., 2019). Such N‐foraging activities of EM hyphae are, however, energy‐ and C‐demanding. Given that EM hyphae depend solely on their host plants for C supply, it would not be surprising if ectomycorrhizal plants have evolved mechanisms to specifically support those of their associated fungal partners that are actively foraging for N.

It is known that plants allocate a greater share of C to resource‐foraging roots (Eissenstat *et al*., 2015; Chen *et al*., 2016; Cheng *et al*., 2016). Recently, Bogar *et al*. (2019) showed in a split‐root experiment with ectomycorrhizal *Pinus muricata* seedlings that plants directed recent photosynthates preferentially to roots and mycorrhizas that had access to an additional nitrogen source. This indicates that a similar mechanism to that which supplies resource‐foraging roots with greater C investments (Eissenstat *et al*., 2015; Chen *et al*., 2016; Cheng *et al*., 2016) could also be employed to supply resource‐foraging mycorrhizal hyphae (Bogar *et al*., 2019).

There are two differences, from a plant perspective, between supporting foraging roots and foraging ectomycorrhizal hyphae. The first difference is that the root system of a single tree is usually colonized by many different individual fungal partners (Lang & Polle, 2011), which are heterogeneously distributed at small scales. Even neighbouring root tips can be associated with different mycorrhizal fungal individuals or species (Lang *et al*., 2011; Gorka *et al*., 2019). In order to support a specific N‐foraging mycorrhiza, plants would thus need to be able to allocate C at a very small scale, by distinguishing among different mycorrhizal root tips along a fine root. The second differences is that, in contrast to the strategy of supporting foraging roots, the unconditional support of foraging mycorrhizal hyphae is naturally linked to the risk of being exploited, as fungi could take the C while keeping the nutrients for themselves (Corrêa *et al*., 2012; Näsholm *et al*., 2013). It has been suggested that such cheating strategies of ectomycorrhizal fungi could be species‐specific (Pena & Polle, 2013) and also dependent on N availability (Albarracín *et al*., 2013).

The risk of being exploited could be lowered if C for nutrient exchange between plants and EM fungi were regulated by reciprocal rewards, that is, if C transfer to foraging fungal hyphae were dependent on the nutrients the plant receives in return (Kiers *et al*., 2011; Fellbaum *et al*., 2014; Walder & van der Heijden, 2015). It has been suggested that mycorrhizal plants and their fungal partners have evolved strategies for the recognition of more beneficial symbiotic partners, allowing them to direct more resources to partners that deliver more in return (Steidinger & Bever, 2014; Werner *et al*., 2014; Wyatt *et al*., 2014). The potential existence of such a control has been shown for the arbuscular mycorrhizal symbiosis, where in simplified experimental systems, plant roots delivered more C to fungal partners that provided more nutrients (phosphorus (P) or N), and vice versa (Bever *et al*., 2009; Fellbaum *et al*., 2011, 2014; Kiers *et al*., 2011, 2016). However, in this regard much less is known about the ectomycorrhizal symbiosis.

The control over nutrient transfer between plants and mycorrhizal fungi takes place at the symbiotic interface – that is, within the Hartig net in the ectomycorrhizal symbiosis. A key role in this control is ascribed to the symbiosis‐specific expression of carbon and nutrient transporter genes of both partners (López *et al*., 2008; Nehls, 2008; Hortal *et al*., 2017). It is possible that such a symbiosis‐controlled C transfer to a certain mycorrhizal partner at a particular location in the root system creates a local C sink for the plant, drawing further plant C to that location. However, very little is known about the mechanisms of resource exchange at the cellular scale at the ectomycorrhizal symbiotic interface. One reason for this is that standard methods used to trace isotopically labelled elements through the plant–soil system (e.g. isotope‐ratio mass spectrometry) are unable to distinguish between plant and fungal tissue within mycorrhizas. Nanoscale secondary ion mass spectrometry (NanoSIMS) has the potential to overcome this problem by enabling mapping of the distribution of multiple isotopes and elements at a spatial resolution of down to 50 nm, which allows tracking of photoassimilated C and fungal‐derived N at a subcellular scale in mycorrhizal tissue (Kaiser *et al*., 2015).

The aim of this study was to investigate whether plants allocate more of their recently photoassimilated C to fungal partners that deliver more N in the ectomycorrhizal symbiosis of European beech (*Fagus sylvatica*), and if so, at which spatial scale such a ‘plant response’ occurs. Specifically, we asked the following questions: (i) Are beech trees allocating recent photosynthates to parts of the root system associated with N‐delivering mycorrhizal fungi, and if so, is this happening only on a coarse scale (i.e. between larger parts of the root system) or also on a finer scale (i.e. among parts of fine roots and individual mycorrhizal root tips)? (ii) How are recently assimilated C and fungus‐delivered N compounds spatially distributed at the cellular scale in the ectomycorrhizal tissue? (iii) Is there any evidence for a mechanism for a targeted exchange of C and N at the cellular scale? Are recent photosynthates spatially linked to N recently taken up by mycorrhizal fungi?

We exposed young beech trees (*F. sylvatica*) in split‐root boxes to a ^13^C‐CO_2_ enriched atmosphere, and simultaneously provided a ^15^N labelled nutrient solution to ectomycorrhizal fungi that were associated with one half of the root system. Within 24–48 h after label application we determined the total ^13^C and ^15^N content of roots, root segments (2–4 cm) and individual mycorrhizal root tips via elemental analysis–isotope‐ratio mass spectrometry (EA‐IRMS). Cross‐sections of a mycorrhizal root tip were prepared for analysis with NanoSIMS to visualize the spatial distribution of ^13^C and ^15^N at the cellular and subcellular scales.

## Materials and Methods

### Pot cultivation of *Fagus sylvatica* and their natural ectomycorrhizal fungi

**Fig. 1:**
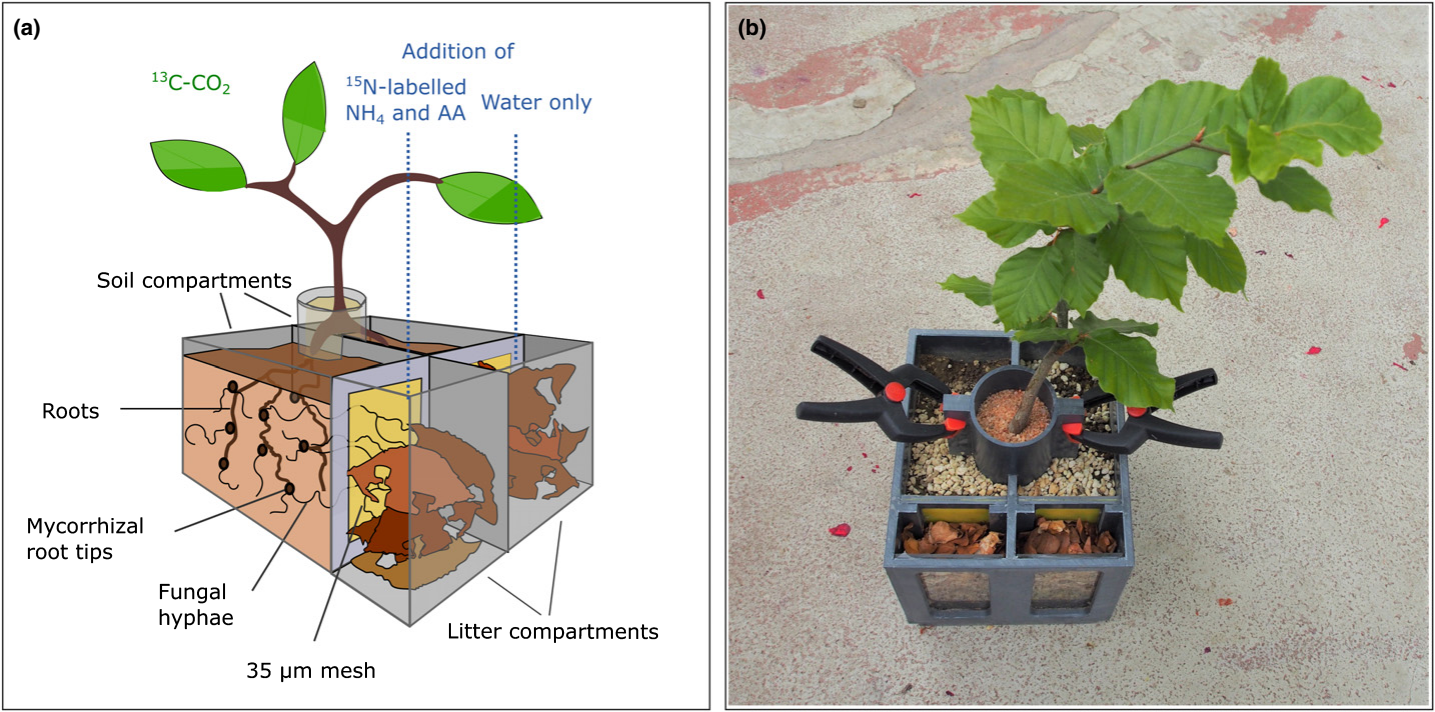
Experimental design of split‐root boxes. Shown is an illustration of the split‐root box setup (a) and a photograph of one of the experimental trees growing in such a box shortly before harvest (b). Plants were grown for *c*. 1 yr in split‐root boxes (155 × 135 × 125 mm) with their root systems divided between two separated compartments, each filled with soil (‘soil compartments’, each 121 × 60 × 125 mm inside). The plant stems were stabilized using a cylinder filled with quartz sand. Each soil compartment was connected to a litter compartment (17 × 60 × 125 mm inside) by a double‐layered 35 µm nylon mesh, which allowed fungal hyphae, but not roots, to grow through. A solid plastic grid was placed in between the mesh layers, which creates an air gap to prevent the exchange of water and solutes between the soil and litter compartments. The two litter compartments were separated by a solid wall from each other, and were filled with beech leaf litter to foster hyphal growth from both root system halves into their respective litter compartments. After 1 yr of growth, we added a ^15^N labelled ammonium chloride (NH_4_) and amino acid (AA) solution to only one of the two litter compartments to provide an additional N source, accessible to one half of the plant's root system via its associated mycorrhiza. Within 24 h of the N addition we exposed the plant's canopies to ^13^C‐labelled CO_2_ using a gas‐tight acrylic glass incubation chamber.

Young (*c*. 3–4‐yr‐old) beech trees (*Fagus sylvatica* L.) colonized by natural mycorrhizal communities were collected from a temperate beech forest (Klausenleopoldsdorf, Lower Austria; stagnic‐gleyic brown earth over flysch sandstone). Twenty‐seven trees were planted into ‘split‐root’ boxes, dividing each plant's root system into two halves growing into distinct ‘soil compartments’ (Fig. 1). Each soil compartment was further connected, via a 35 µm nylon mesh (penetrable by fungal hyphae but not plant roots), to an exclusive ‘litter compartment’ (Fig. 1). The mesh consisted of two layers with a solid plastic grid in between, which created an air gap, preventing water flow from one compartment into the other (Gorka *et al*., 2019). Soil compartments were filled with a mixture of soil (A‐horizon collected from the site, 4 mm sieved) and perlite (soil : perlite ratio of 8 : 1, v/v). Litter compartments (17 × 60 × 125 mm) were filled with beech litter collected from the same site.

Trees were grown for 1 yr (July 2014–June 2015) in the split‐root boxes in an ‘open’ glasshouse under ambient (outdoor) sunlight and temperature and were watered regularly. Examination of selected plant roots under the stereomicroscope showed abundant mycorrhizal colonisation at the time of transfer from the forest to the split‐root boxes, as well as during the experimental harvest 1 yr later (see the supplementary material in a previous article by Gorka *et al*., 2019). Amplicon sequencing of the internal transcribed spacer (ITS) region of extracted DNA showed that mycorrhizal community composition in the litter compartments and rhizospheres of the plants was similar, indicating that mycorrhizal hyphae had grown from the plant roots into the litter compartments (Gorka *et al*., 2019). For more details on the experimental setup see the study by Gorka *et al*. (2019), which presents additional results from the same experiment.

### Double stable isotope (^13^C, ^15^N) pulse labelling

We provided a ^15^N‐labelled N source to mycorrhizal hyphae associated with one half of the root system, but not to the roots directly, by adding 12 ml of a ^15^N‐labelled NH_4_ and amino acid mix to one of the two litter compartments (in eight split‐root boxes). The second litter compartment of each box received water as control. The ^15^N labelled NH_4_ and amino acid mix consisted of ^15^N‐NH_4_Cl (98 at%, 54.5 mg l^−1^, i.e. 1 mM N; Sigma‐Aldrich) and an Algal Amino Acid mixture (U‐^15^N 98 at%; 140 mg l^−1^, *c*. 1 mM N based on an assumed average amino acid molar weight of 140 g mol^−1^; Cambridge Isotope Laboratories, Cambridge, UK), dissolved in water. Twenty‐four hours after the ^15^N labelling, the canopies of these eight trees, plus four trees which only received water on both sides, were exposed to ^13^CO_2_ for 6 h 20 min at *c*. 90 at% (1500 ppm) using a gas‐tight acrylic glass incubation chamber, with which plant canopies can be exposed exclusively to the chamber atmosphere, while keeping soil and litter compartments outside, thereby preventing direct contact between them and the ^13^CO_2_ (for details, see the earlier study by Gorka *et al*., 2019). Gas samples were retrieved from the labelling chamber at regular intervals and analysed for CO_2_ concentration and isotopic ratio using a headspace gas sampler (GasBench II; Thermo Fisher Scientific, Bremen, Germany) interfaced to a continuous flow isotope‐ratio mass spectrometer (Delta Advantage V; Thermo Electron, Bremen, Germany). Four additional planted split‐root boxes were kept without any ^13^C or ^15^N labelling to serve as natural abundance controls. A preliminary experiment had been conducted to determine optimal harvest times, with the aim of harvesting plants as soon as possible after both ^13^C and ^15^N were detectable in plant roots.

### Plant harvest: root segments and root tips

Plants were kept in the dark overnight after the ^13^CO_2_ labelling (to allow further belowground transport of the photoassimilated C) and were harvested over 10 h during the next day (i.e. 19–29 h after the start of the 6 h labelling period, referred to as *c*. 24 h after labelling from now on). Boxes were carefully taken apart and aboveground biomass was clipped and stored before excavation of the belowground parts. Roots were taken out of each soil compartment, rinsed with tap water and kept moist in water containers for further analysis. Each root system half was further separated into 4–5 approximately equally sized segments, from which mycorrhizal root colonisation was roughly estimated under stereomicroscopes (Gorka *et al*., 2019). A number of individual branched root tips (*c*. 3–5 mm, for an example see Supporting Information Fig. S1), with each branch clearly representing the same ‘morphotype’ (established by visual identification under the microscope; Agerer, 1989), were sampled from each root system. Each of these branched root tips was further separated into three smaller pieces (‘individual root tips’), which were stored either at −20°C (for ITS sequencing), air dried (for bulk isotope analysis via EA‐IRMS) or in liquid N_2_ after ultra‐rapid cooling by plunge freezing in liquid propane (for NanoSIMS). Litter compartments did not contain any roots, proving that roots had been successfully prevented from growing into these compartments by the mesh barrier. Litter compartments were also harvested and analysed, the results of which can be found in the earlier study by Gorka *et al*. (2019).

### Bulk isotope analysis of root segments and root‐tips

Isotopic ratios (^13^C : ^12^C and ^15^N : ^14^N) of individual mycorrhizal root tips were analysed using an elemental analyzer (EA 1110 elemental analyzer, CE Instruments, Wigan, UK) coupled to an isotope‐ratio mass spectrometer (IRMS, Finnigan MAT DeltaPlus; Fisher Scientific, Vienna, Austria). Root segments were ground, and their bulk isotope ratios were measured by IRMS. Isotopic enrichment was calculated as the difference in relative isotopic enrichment between labelled and unlabelled control samples (i.e. as at% excess (APE)).

### Sequencing of the internal transcribed spacer region

DNA was extracted from the mycorrhizal root tip taken from the same branch as the root tip used for the NanoSIMS analysis using a phenol‐chloroform protocol based on physical shearing of the cells by four bead beating steps (adapted from the method described by Angel, 2012). DNA was eluted in 30 µl Low TE buffer and stored at −80°C before sequencing. The fungal ITS1 region was amplified via polymerase chain reaction (PCR) with the primer pair ITS1F (5′ CTT GGT CAT TTA GAG GAA GTA A 3′) (Gardes & Bruns, 1993) and ITS2 (5′ GCT GCG TTC TTC ATC GAT GC 3′, used as the reverse primer) (White *et al*., 1990). Amplicons were prepared for sequencing on the Illumina MiSeq platform (Illumina, San Diego, CA, USA) using the multiplexed barcoded amplicon approach, as described by Herbold *et al*. (2015). For further details see the earlier study by Gorka *et al*. (2019). The sequence data were deposited in the National Center for Biotechnology Information (NCBI) Short Read Archive under study accession no. PRJNA606050.

### Preparation of mycorrhizal root tips for nanoscale secondary ion mass spectrometry analysis

To minimize potential losses of labile compounds such as sugars and amino acids, a low‐temperature freeze substitution method was used (Kilburn & Clode, 2014; Kaiser *et al*., 2015). Mycorrhizal root tips were dissected and immediately frozen in liquid propane (−183°C) using a plunge freezer (EM CPC; Leica Microsystems, Vienna, Austria). Samples were freeze‐substituted in 10% acrolein in diethyl ether in a −80°C freezer for 3 wk followed by a controlled warm‐up to room temperature (1.5°C h^−1^) in an automated freeze substitution system (AFS2; Leica Microsystems). Subsequently, they were washed in diethyl ether, and embedded and infiltrated in pure LR‐white resin (following a modified protocol after Kilburn & Clode, 2014; Kaiser *et al*., 2015). Heat polymerisation was performed at 40°C under vacuum. Cross‐sections, 1 µm in thickness, were cut with glass knives using an ultramicrotome UC7 (Leica Microsystems), mounted on glass slides and subsequently stained with toluidine blue. Suitable analysis areas for subsequent NanoSIMS imaging were selected using light microscopy at ×40 magnification (Leica CTR 6500; Leica Microsystems). Consecutive sections were deposited onto indium tin oxide (ITO) coated glass slides (7.1 × 7.1 × 1.1 mm; Praezisions Glas & Optik GmbH, Iserlohn, Germany) and sputter‐coated with a gold/palladium (Au : Pd; 80 : 20) layer of 40 nm (nominal thickness) to prevent electric charging through the NanoSIMS measurement process.

### Nanoscale secondary ion mass spectrometry analysis

**Fig. 2:**
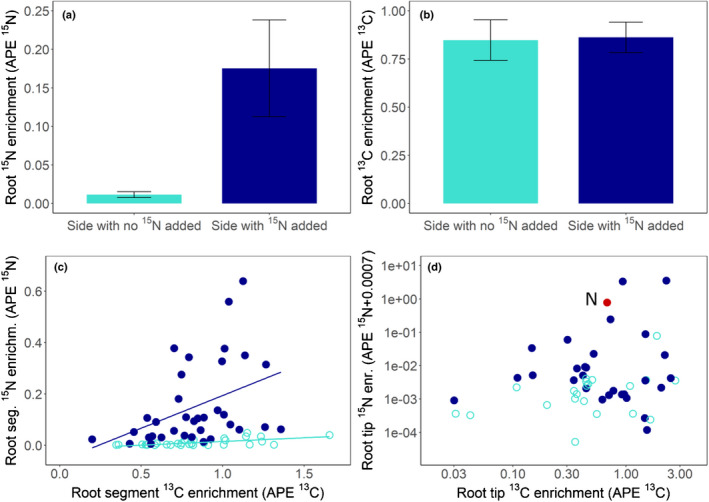
^13^C and ^15^N enrichments (at% excess, APE) of root system halves (a, b), individual fine root segments (c) and mycorrhizal root tips (d) from six beech trees grown in split‐root boxes. Mycorrhizal fungi associated with one half of the root system had access to an additional source of ^15^N‐labelled nitrogen (dark blue bars, closed circles) whereas mycorrhizas associated with the other half of the root system had no access to the labelled nitrogen source (light blue bars, open circles), but could have received it through internal redistribution within the plant. Trees were exposed to ^13^C‐labelled CO_2_ 24 h after ^15^N addition and 24 h before harvest. (a, b) Bars show the mean ^13^C and ^15^N enrichment of the root system halves (calculated as the weighted mean of all measured segments of each root system half) of six plant replicates (error bar, ± SE, *n* = 6). (c) The lines depict linear regressions of carbon and nitrogen isotope enrichment in individual root segments (dark blue line: N‐amended side, *P* = 0.014, *R* ^2^ = 0.1416, *n* = 35; light blue line: N‐free side, *P* < 0.0001, *R* ^2^ = 0.39, *n* = 32). (d) ^13^C and ^15^N enrichments of individual root tips of N‐amended and unamended sides. Root tip data in (d) are represented on logarithmic scales. No significant correlation was found for this relationship. The specific root tip analysed using nanoscale secondary ion mass spectrometry (NanoSIMS) is marked in red (N).

Nanoscale secondary ion mass spectrometry elemental and isotope analysis was performed using a NanoSIMS 50L instrument (Cameca, Gennevilliers, France) at the University of Vienna. Measurements were made using a 16 keV Cs^+^ primary ion beam with simultaneous detection of seven secondary ion species, ^13^C^−^, ^12^C^−^, ^13^C^12^C^−^, ^12^C^12^C^−^, ^12^C^15^N^−^, ^12^C^14^N^−^, ^31^P^−^, as well as secondary electrons. Sixteen images were acquired on a cross‐section of one ^13^C and ^15^N labelled mycorrhizal root tip (Fig. 2) in a mosaic array, each measuring 70 × 70 µm (with a 20 µm overlap, yielding a total image size of 200 × 200 µm). Acquiring consecutive and partly overlapping NanoSIMS images from the same sample initially led to a bias in measured ^15^N signatures due to adsorption of N_2_ with a natural isotopic abundance in areas that had already been measured; this was solved by additional pre‐sputtering between acquisition of individual images (Notes S1; Figs S2, S3). In addition, four images encompassing all mycorrhizal tissue types were acquired on a cross‐section of a mycorrhizal root tip taken from a tree which had not received any ^13^C or ^15^N, as a natural abundance control. Images were recorded as multilayer image stacks with a 512 × 512 pixel image resolution and *c*. 70 nm physical resolution (probe size), with a total per‐pixel dwell time of 13.5 ms. For more technical detail on the NanoSIMS measurements and data evaluation see Methods S1.

### Nanoscale secondary ion mass image data evaluation

Image data were processed utilizing the OpenMIMS plugin (Center for Nano Imaging, https://nano.bwh.harvard.edu/MIMSsoftware) for the image analysis software ImageJ (National Institutes of Health, Bethesda, MD, USA). Carbon isotope composition images displaying the ^13^C/(^12^C+^13^C) isotope fraction, designated at%^13^C, were inferred from the C_2_^−^ secondary ion signal intensity distribution images via per‐pixel calculation of ^13^C^12^C^−^/(2·^12^C^12^C^−^+^13^C^12^C^−^) intensity ratios. Similarly, nitrogen isotope composition images displaying the ^15^N/(^14^N+^15^N) isotope fraction, designated at%^15^N, were inferred from the ^12^CN^−^ secondary ion signal intensity maps via per‐pixel calculation of ^12^C^15^N^−^/(^12^C^15^N^−^+^12^C^14^N^−^) intensity ratios. Atomic percentage excess values were determined relative to the ^13^C and ^15^N isotope fractions (at%) measured on an unlabelled control sample (Table 1). CN^−^ secondary ion maps were used for visualization of cellular structures. Taking into account the dual stable isotope labelling of the samples, the total CN^−^ signal intensity was obtained via per‐pixel calculation of ^12^C^14^N^−^(1+R^13^C/^12^C) + ^12^C^15^N^−^(1+R^13^C/^12^C) values, where R^13^C/^12^C refers to the carbon isotope ratio, inferred from the C_2_^−^ signal intensities (R^13^C/^12^C =^13^C^12^C^−^/(2·^12^C^12^C^−^)). Overlay images, combining structural with chemical information, were created using Adobe Photoshop CS6. Multi‐tile mosaic images were assembled with Adobe InDesign CS6.

**Table 1:** Mean stable isotope signatures (at%^13^C and ^15^N) of individual regions of interest (ROIs) defined for different ectomycorrhizal tissue types (left‐most column) across NanoSIMS images from cross‐sections of an ectomycorrhizal root tip of *Fagus sylvatica* that had been exposed to ^13^CO_2_ and ^15^N labelling, and from one which had not been exposed (unlabelled control).

		Label									Control				
		*n*	at%^13^C				at%^15^N				*n*	at%^13^C		at%^15^N	
			Mean	RSD	Min	Max	Mean	RSD	Min	Max		Mean	RSD	Mean	RSD
CW	HE	425	1.29	0.46	1.05	5.11	3.93	1.29	0.48	21.30	20	1.08	0.01	0.37	0.02
	HM	371	1.14	0.06	1.06	1.77	3.27	0.43	0.82	7.46	29	1.07	0.01	0.37	0.02
	HN	322	1.22	0.11	1.08	2.53	3.55	0.41	0.92	8.38	104	1.07	0.01	0.37	0.03
	PC	82	1.17	0.06	1.09	1.49	2.15	0.46	0.92	5.66	34	1.07	0.01	0.37	0.03
	E	34	1.47	0.44	1.12	4.22	1.70	0.64	0.76	4.10	16	1.06	0.00	0.36	0.03
	VT	88	1.67	0.81	1.13	6.68	2.39	0.35	0.67	3.88	76	1.07	0.01	0.37	0.04
L	HE	217	1.16	0.14	0.98	2.72	6.10	0.48	0.31	16.86	11	1.06	0.01	0.37	0.04
	HM	225	1.12	0.05	1.03	1.41	5.54	0.44	0.55	11.77	20	1.06	0.00	0.37	0.04
	HN	124	1.20	0.14	1.03	2.10	4.19	0.60	0.53	11.63	53	1.07	0.01	0.37	0.04
	PC	116	1.09	0.03	1.05	1.25	0.98	0.85	0.41	3.21	45	1.06	0.00	0.38	0.02
	E	24	1.10	0.05	1.07	1.30	1.12	0.76	0.47	2.57	10	1.07	0.00	0.37	0.02
	VT	61	1.14	0.10	1.06	1.59	1.56	0.86	0.44	4.23	48	1.06	0.01	0.37	0.06
Resin		117	1.07	0.02	0.99	1.22	0.47	0.49	0.19	1.39	25	1.06	0.00	0.38	0.02

CW, cell wall; E, endodermis; HE, extended hyphae; HM, hyphae mantle; HN, hyphae Hartig net; L, lumen; *n*, number of analysed ROIs; PC, plant cortex; RSD, relative standard deviation; VT, vascular tissue.

Approximately 2200 regions of interest (ROIs) were defined across all NanoSIMS images and were annotated to plant and fungal tissues on the basis of the secondary electron signal intensity maps, distinguishing between cell wall and lumen within and between cells, respectively (ImageJ/OpenMIMS). Plant and fungal tissue was further classified into subcategories, from the outside to the inside of the mycorrhizal root tip as follows: external (i.e. emanating) hyphae (HE), hyphae mantle (HM), hyphae Hartig net (HN), parenchyma plant cortex (PC), endodermis (E) and vascular tissue (VT). Average secondary ion signal intensities were extracted from each area and used for calculation of the ROI specific carbon and nitrogen isotope compositions.

### Statistics

Statistical calculations were performed in R v.3.3.2 (R Core Team, 2016); linear and segmented linear regression analyses were employed, the latter using the package segmented (Muggeo, 2003, https://cran.r‐project.org/web/packages/segmented/index.html). Graphs were plotted using the package ggplot2 (Wickham, 2016; http://ggplot2.org/). Normal distribution was tested for using the Kolmogorov–Smirnov test. Variance homogeneity was tested using the *F*‐test. Since the data did not meet these requirements, a generalized linear model (GLM) was applied, combined with the post‐hoc Tukey test in the past (Paleontological statistics) software package (Hammer *et al*., 2001). Means were considered to be significantly different from each other for *P* < 0.05. Line‐scan analysis (see Fig. 8) was performed with a resolution of 3 pixels per data point.

## Results

### Distribution of photoassimilated carbon and fungus‐delivered nitrogen at the root system scale

Our experimental setup allowed tracing of the allocation of recent photosynthates through the root system at different spatial scales. At the largest scale we distinguished between root system halves that received additional N via their mycorrhizal partners, and those that did not (Fig. 2a,b). Root segments and root tips of the ‘N‐amended’ side of the box (i.e. those connected to litter compartments which had received ^15^N‐labelled compounds) were significantly and highly enriched in ^15^N (root segments up to 0.6 at%^15^N excess, root tips up to 3.5 at%^15^N excess, Fig. 2). Root segments and root tips from the ^15^N‐unamended side, by contrast, were only slightly and occasionally enriched above natural abundance levels (Fig. 2). There was no difference in the overall ^13^C enrichment (at%^13^C excess) between the N‐amended and N‐unamended sides of the root system at that scale (Fig. 2b). However, we found a significant correlation between ^13^C and ^15^N enrichment of cm‐sized individual root segments in each side of the pot (Fig. 2c). The slope of the regression was steeper for the side of the plant that received ^15^N directly via their associated fungal hyphae (^15^N_exc_ = 0.25 × ^13^C_exc_ – 0.06, *P* = 0.014, *R* ^2^ = 0.1416), as root segments were generally more highly enriched in ^15^N (APE ^15^N: mean = 0.15, med = 0.09, SD = 0.16, SE = 0.027, *n* = 35). Root segments of the unamended side were, on average, much less enriched in ^15^N (mean = 0.01, med = 0.003, SD = 0.013, SE = 0.0023, *n* = 32). However, the correlation between the ^13^C and ^15^N enrichment of root segments in the ^15^N unamended side of the plant, which presumably received ^15^N only by internal translocation within the plant, was more significant and explained more of the variation compared to that of the ^15^N‐amended side (^15^N_exc_ = 0.029 × ^13^C_exc_ – 0.013, *P* < 0.0001, *R* ^2^ = 0.39; Fig. 2c).

The distribution of ^15^N was more heterogeneous across individual root tips compared to root segments on the N amended sides (mean = 0.28, SD = 0.88, SE = 0.16, *n* = 29); many samples were only slightly enriched, and a few were highly enriched (min = 0.0, max = 3.5, med = 0.003; Fig. 2d). There was no significant correlation between ^13^C and ^15^N across root tips of the N‐amended sides; however, the four root tips which showed a ^15^N enrichment of > 0.1 APE were also in the upper range of measured ^13^C enrichments (i.e. between 0.68 and 2.28 at%^13^C excess). On the ^15^N unamended sides only 11 out of 21 root tips were labelled above the natural abundance level (mean = 0.004, med = 0.001, max = 0.07) and there was no correlation between ^13^C and ^15^N. Similar to root segments, there was no difference in the overall mean of ^13^C enrichment of root tips between N‐amended and ‐unamended sides (Fig. 2d).

For analysis of ^13^C and ^15^N distributions at a smaller scale (i.e. in the tissue within a single mycorrhizal root tip) we selected one of the root tips with above‐average ^15^N enrichment (0.777 APE ^15^N and 0.682 APE ^13^C, Fig. 2d), under the assumption that it actively participated in the nutrient delivery from the litter compartment to the plant.

Internal transcribed spacer sequencing of the adjacent branch of this root tip revealed that the fungal community associated with this root tip was dominated by two fungal operational taxonomical units (OTUs) belonging to the genus *Thelephora* (constituting 35% and 23% relative abundance, with both OTUs having the identifier SH1502189.08FU according to the UNITE database; similar sequences of the same identifier have often been classified as *Thelephora terrestris*). All other OTUs occurred at < 1.3% relative abundance, indicating that this specific root tip was colonized by *T. terrestris*.

### Heterogenous distribution of photoassimilated carbon and fungus‐delivered nitrogen at the cellular scale within a mycorrhizal root tip

**Fig. 3:**
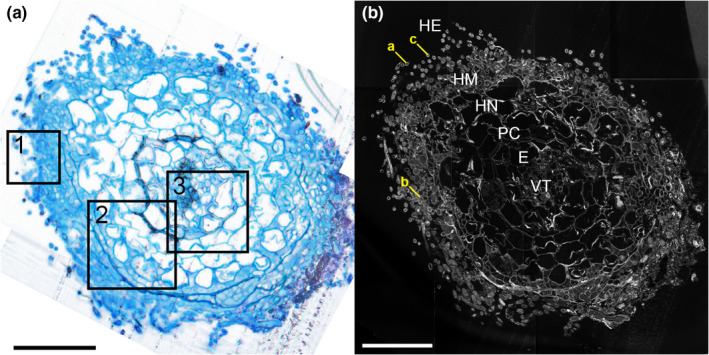
(a) Light microscopy image of a cross‐section of an ectomycorrhizal root tip of beech (*Fagus sylvatica*) associated with fungi from the genus *Thelephora*, stained with toluidine blue. Squares refer to the areas shown in Fig. 4. (b) Total CN^−^ secondary ion signal intensity distribution image, visualizing the cellular structure of a consecutive section analysed using NanoSIMS. E, endodermis; HE, extended hyphae; HM, mantle hyphae; HN, Hartig net; PC, plant cortex; VT, vascular tissue. Labels ‘a’, ‘b’ and ‘c’ indicate external hyphae selected for linescan analysis (Fig. 8). Bars, 50 µm.

**Fig. 4:**
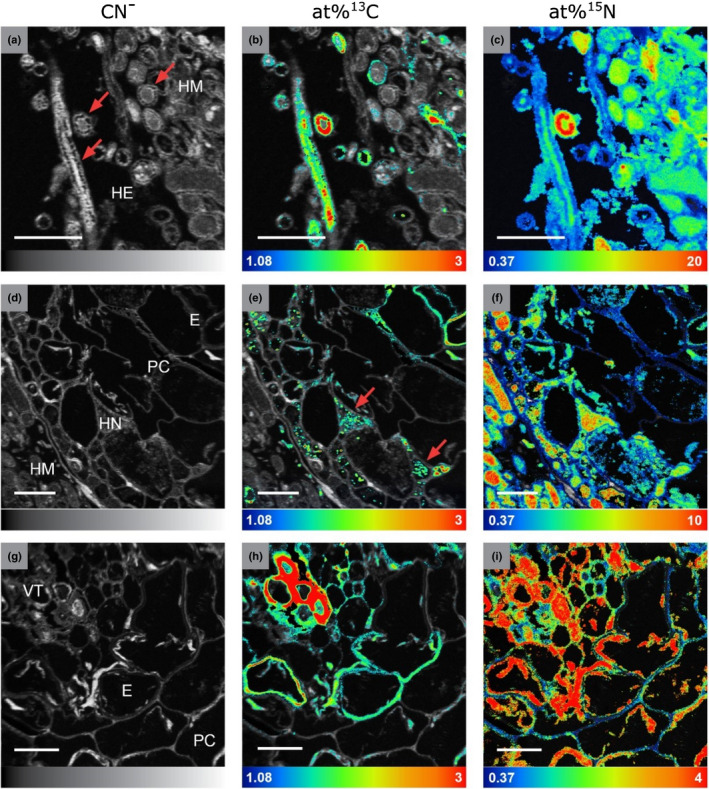
Nanoscale secondary ion mass spectrometry (NanoSIMS) images from three selected areas (indicated in Fig. 3) of the root tip cross‐section (shown as an assembled mosaic image in Figs 5, 6). Isotopic label content is displayed as at%^13^C and at%^15^N. Colour scales range from the natural isotope abundance (determined on an unlabelled control) to 3 at%^13^C and 20 at%^15^N. Image series from left to right: Total CN^−^ signal intensity distribution, visualizing the cellular structure (a, d, g); overlay of the CN^−^ and at%^13^C distribution images (b, e, h), overlay of the CN^−^ and at%^15^N images (c, f, i). Colours depicting the natural abundances of ^13^C and ^15^N were omitted in the overlay images to allow for a better visualisation of the structural information (NanoSIMS images displaying solely at%^13^C and at%^15^N information are provided in Supporting Information Figs S6, S7, with colour‐blind friendly versions in Figs S8, S9). E, endodermis; HE, extended hyphae; HM, mantle hyphae; HN, Hartig net; PC, plant cortex; VT, vascular tissue. Red arrows indicate (a) the septate hyphae structure and (e) a punctual ^13^C enrichment in Hartig net hyphae. Bars, 10 µm.

**Fig. 5:**
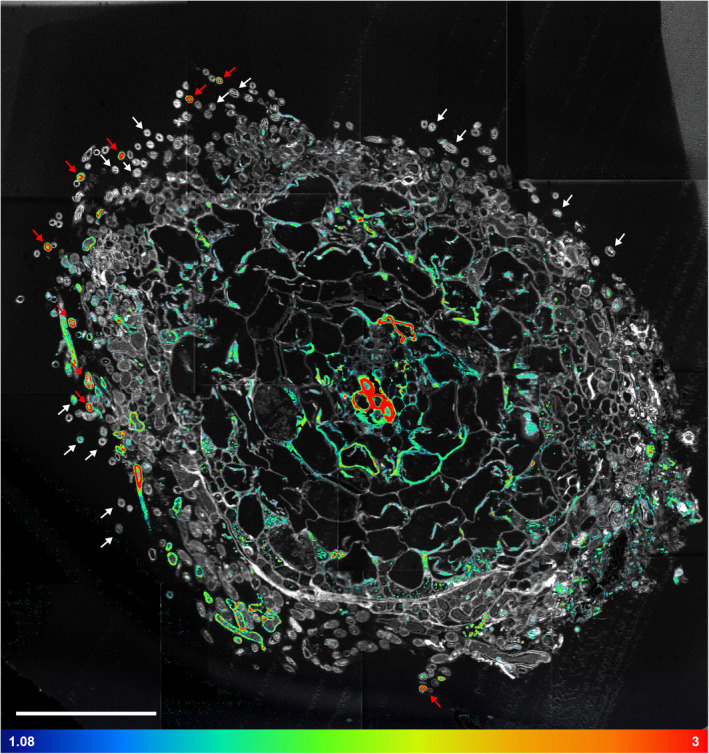
Nanoscale secondary ion mass spectrometry (NanoSIMS) visualization of the spatial distribution of ^13^C enrichment within an ectomycorrhizal root tip of beech (*Fagus sylvatica*) associated with fungi from the genus *Thelephora* 24 h after the plant has been exposed to a ^13^C‐CO_2_ atmosphere and 48 h after the fungi accessed a ^15^N‐labelled N source. Shown is an overlay of the total CN^−^ secondary ion signal intensity distribution image (Supporting Information Fig. S10) and the corresponding ^13^C label distribution image (Fig. S6) acquired on a cross‐section of the sampled root tip. The picture consists of 16 individual images (each 50 × 50 µm), assembled as a mosaic. The isotopic label content is presented as at%^13^C, displayed on a false colour scale ranging from the natural abundance value (dark blue, determined on an unlabelled control) to 3 at%^13^C (red). For a better visualization of areas enriched in ^13^C, colouring representing the natural abundance of the isotope is omitted in the overlay image (NanoSIMS images displaying solely at%^13^C information are provided in Fig. S6, with a colour‐blind friendly version in Fig. S8). White arrows indicate external hyphae exhibiting low ^13^C and ^15^N enrichment, which typically show ^15^N in the centre of the hyphal cross‐section (decoupled from ^13^C enrichment), whereas red arrows indicate those with overall high ^13^C and ^15^N enrichment, where ^13^C and ^15^N are typically co‐localized in the outer ring of a hyphal cross‐section (cf. Fig. 8). Bar, 50 µm.

**Fig. 6:**
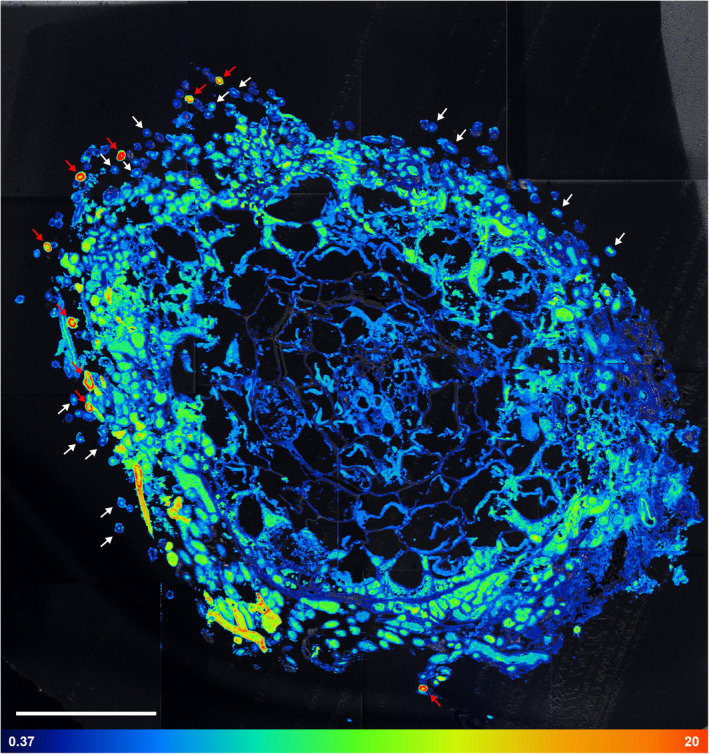
Nanoscale secondary ion mass spectrometry (NanoSIMS) visualization of the spatial distribution of ^15^N enrichment within an ectomycorrhizal root tip of beech (*Fagus sylvatica*) associated with fungi from the genus *Thelephora* 24 h after the plant has been exposed to a ^13^C‐CO_2_ atmosphere and 48 h after the fungi accessed a ^15^N‐labelled N source. Shown is an overlay of the total CN^−^ signal intensity distribution image (Supporting Information Fig. S8) and the corresponding ^15^N label distribution image (Fig. S7), acquired on a cross‐section of the sampled root tip. The picture consists of 16 individual images, assembled as a mosaic (each 50 × 50 µm). The isotopic label content is presented in terms of at%, displayed on a false‐colour scale ranging from the natural abundance value (dark blue, determined on an unlabelled control) to 20 at%^15^N (red). For a better visualization of areas enriched in ^15^N, colouring representing the natural abundance of the isotope is omitted in the overlay image (NanoSIMS images displaying solely at%^15^N information are provided in Fig. S7, with a colour‐blind friendly version in Fig. S9). White arrows indicate external hyphae exhibiting low ^13^C and ^15^N enrichment, which typically show ^15^N in the centre of the hyphal cross‐section (decoupled from ^13^C enrichment), whereas red arrows indicate those with overall high ^13^C and ^15^N enrichment, where ^13^C and ^15^N are typically co‐localized in the outer ring of a hyphal cross‐section (cf. Fig. 8). Bar, 50 µm.

Our NanoSIMS analysis revealed a snapshot of the *in situ* spatial distribution of isotopically enriched plant photosynthates and fungus‐delivered nitrogen compounds (^13^C, ^15^N; Figs 3, 4, 5, 6) in the cross‐section of an ectomycorrhizal root tip formed by *F. sylvatica* and *T. terrestris*, 24 h after photosynthesis and 48 h after the ^15^N‐labelled N source was added to the fungal compartment. On average, the isotopic enrichment within ROIs ranged from 0 to 20.9 at%^15^N excess and 0 to 5.61 at%^13^C excess. The highest ^15^N content was observed in external hyphae (up to 21 at% in cell walls, CW; and 16 at% in lumen, L), followed by fungal cells of the mantle and the Hartig net (Table 1). The plant tissue inside this root tip was also significantly enriched in ^15^N, with up to 4.23 at%^15^N in vascular tissue and epidermal cells, and up to 5.66 at%^15^N in plant cortex cells (Figs 4, S4; Table 1), thereby demonstrating that ^15^N has been passed on from the fungi to the plant. The plant cells of the vascular tissue showed the highest ^13^C content (6.68 at%), followed by external hyphal cells (up to 5.11 at%), which exhibited higher maxima than any of the ROIs from the plant cells of the endodermis or plant cortex. Fungal cells showed, on average, a similar ^13^C content to plant cortex cells.

There was a clearly heterogeneous distribution of ^13^C and ^15^N, with strong local enrichments, especially in hyphae emanating from the mantle into the soil (‘external hyphae’, HE; Figs 5, 6). These few extraordinary hyphae (*c*. 10 out of 200) showed by far the highest relative isotopic enrichments (max. 5.11 at%^13^C, 21.3 at%^15^N) of all fungal tissues (Fig. 3b,c). By contrast, the majority of hyphae showed only slight ^13^C enrichment, with 75% of all defined ROIs being within the lowest 2.1% of all measured ^13^C enrichment values (< 0.12 at%^13^C excess). Only 3.4% and 0.09% of all measured ROIs showed no significant enrichment in ^13^C and ^15^N, respectively.

A closer visual inspection of the 16 NanoSIMS images taken from the cross‐section of the mycorrhizal root tip also revealed interesting details regarding the spatial distribution of labelled C and N at the sub‐cellular scale (Figs 4, 5, 6). Especially in fungal cells of the Hartig net, ^13^C‐labelled compounds derived from recent photosynthesis appeared often as collections of ‘droplets’ inside individual fungal cells (Figs 4e, 5). This pattern is less visible in the fungal mantle cells, which are also overall less enriched in ^13^C, and is not visible in the external hyphae. The CN^−^ secondary ion signal intensity distribution image, which displays structural details, reveals that the outer ring or ‘cell walls’ of cross‐sectioned external hyphae is/are characterized by a septate‐like structure (Fig. 4a). Interestingly, these outer cell areas often carry particularly high co‐enrichments of ^15^N and ^13^C, as visible in the overlay images (Figs 4b,c, 5, 6).

### Strong spatial correlation between recent photosynthates and fungus‐delivered nitrogen at cellular scales

**Fig. 7:**
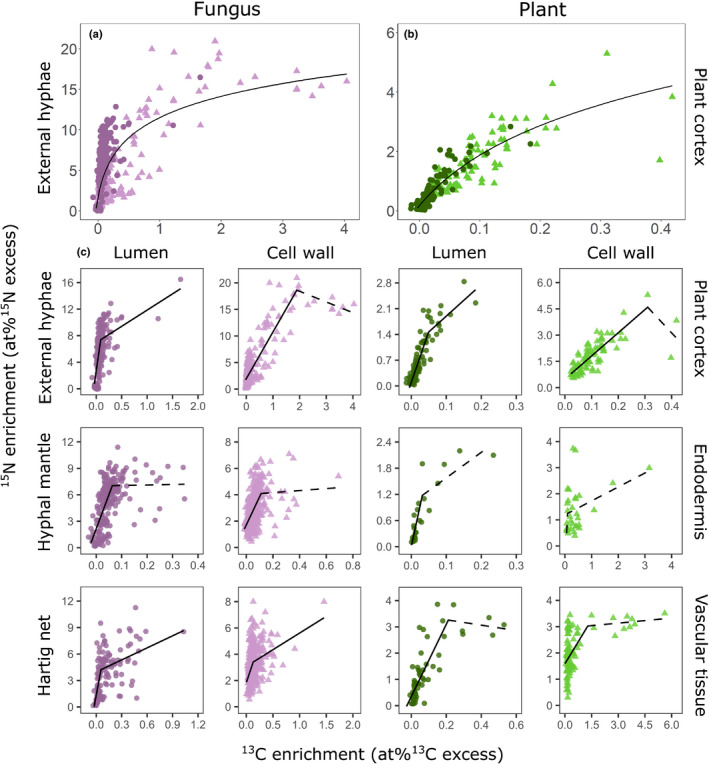
Spatial correlation of relative ^13^C and ^15^N enrichment in plant (green) and fungal (purple) tissues. Each data point represents ^13^C and ^15^N enrichment (in at% excess, APE) of one region of interest (ROI) obtained from NanoSIMS images (Supporting Information Figs S6, S7) of a complete cross‐section of an ectomycorrhizal root tip of beech (*Fagus sylvatica*). Regions of interest were grouped according to tissue type (fungus: external hyphae, hyphal mantle, Hartig net; plant: plant cortex, endodermis, vascular tissue). For each tissue type ROIs were further categorized into lumen (circles) and cell walls (triangles). (a, b) Overview correlations combining lumen and cell wall ROIs of plant cortex and external hyphae, respectively. Lines represent the fit of a logarithmic regression model (fungi: *R* ^2^ = 0.51, *P* < 2.2e^−^^16^; plant: *R* ^2^ = 0.75, *P* < 2.2e^−^^16^). (c) Segmented linear regression analyses for each tissue type. Significant correlations are depicted as solid lines; nonsignificant correlations depicted as dashed lines. Breakpoints of regressions (in c) are located at the kink of each line. Coefficients of determination (*R* ^2^), significance values (*P*) and slopes of correlations depicted in (c) are provided in Table 2. The axis scales are optimized for the data ranges of each correlation; correlations with fixed axis scales for better comparisons among tissue types are provided in Fig. S5.

We found a strong spatial correlation (Spearman rank correlation, *r*_s_fungi_ = 0.45, *P* < 0.0001, *r*_s_plant_ = 0.81, *P* < 0.0001) between ^13^C and ^15^N enrichment across 2090 ROIs defined from plant and fungal tissues in 16 individual NanoSIMS images obtained from one root tip cross‐section (Fig. 7). There is a strong linear relationship between ^15^N and ^13^C at low ^13^C enrichment, but only up to a certain threshold of ^13^C and ^15^N, after which there is either no longer a significant correlation or a correlation with a slope that is much less steep (Fig. 7; Table 2). We performed a model selection procedure based on the log likelihood information criteria (AIC/BIC), where a breakpoint regression model was chosen over log transformation models of ^13^C and/or ^15^N (Table S1). The regression slope breakpoint occurs at lower ^13^C enrichment in plant tissue than fungal tissue, except for the plant vascular tissue (Table 2). We found different trends in cell walls and lumen, with the relationship at high enrichment levels being mainly characterized by strong ^13^C labelling in cell walls.

**Table 2:** Segmented linear regression analysis of isotopic enrichment (at% excess, APE ^13^C and ^15^N) of regions of interest (ROIs) of distinct tissue types measured by nanoscale secondary ion mass spectrometry (NanoSIMS) on a cross‐section of an ectomycorrhizal root tip of *Fagus sylvatica* shortly (1 and 2 d for ^13^C and ^15^N, respectively) after exposure to ^13^CO_2_ and ^15^N labelling.

		Segmented linear model								
		Total			Low			High		
		BP	SE	*R* ^2^	*R* ^2^	*a*	*P*	*R* ^2^	*a*	*P*
Fungi	CW	1.93	0.096	0.65	0.54	7.77	***	0.45	−1.50	*
	L	0.06	0.005	0.39	0.30	64.09	***	0.05	3.27	***
Plant	CW	0.14	0.016	0.28	0.18	9.94	***	0.09	0.32	***
	L	0.11	0.01	0.76	0.62	20.09	***	0.10		ns
HE	CW	1.493	0.084	0.78	0.62	8.86	***	0.04		ns
	L	0.088	0.01	0.48	0.33	53.09	***	0.21	4.90	***
HM	CW	0.111	0.013	0.28	0.22	21.42	***	0.00		ns
	L	0.063	0.006	0.45	0.36	77.51	***	0.00		ns
HN	CW	0.13	0.024	0.18	0.07	12.13	***	0.10	2.54	***
	L	0.056	0.013	0.40	0.27	52.64	***	0.12	4.59	**
PC	CW	0.311	0.112	0.69	0.71	13.54	***	Na		
	L	0.049	0.006	0.83	0.71	27.24	***	0.41	8.32	*
E	CW	0.33	0.658	0.18	0.17	21.95	ns	0.26		ns
	L	0.031	0.009	0.81	0.42	23.00	*	0.40		ns
VT	CW	1.283	0.595	0.32	0.07	1.12	*	0.08		ns
	L	0.212	0.037	0.70	0.54	13.76	***	0.07		ns

*a*, regression slope; BP, estimated breakpoint of segmented linear regression; CW, cell wall; E, endodermis; HE, extended hyphae; HM, hyphae mantle; HN, hyphae Hartig net; L, lumen; *P,* significance level (*, *P* < 0.05; **, *P* < 0.01; ***, *P* < 0.001; ns, not significant; na, not applicable); PC, plant cortex; *R* ^2^, coefficient of determination; SE, standard error; VT, vascular tissue.

### Sub‐cellular spatial distribution of photoassimilates and nitrogen in external hyphae

**Fig. 8:**
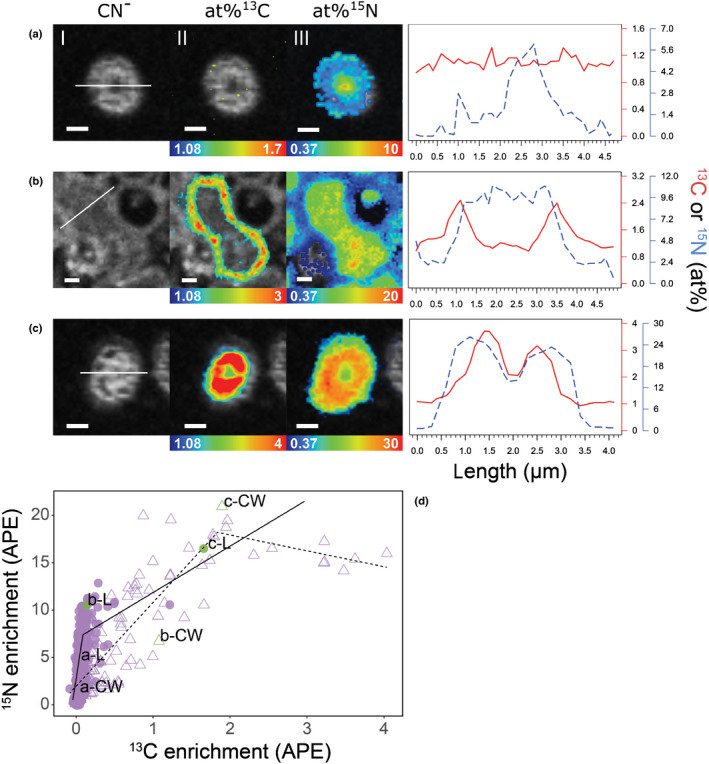
^13^C and ^15^N distribution pattern in individual hyphae emanating from an ectomycorrhizal root tip of beech (*Fagus sylvatica*) associated with fungi from the genus *Thelephora* 24 h after the plant has been exposed to a ^13^C‐CO_2_ atmosphere and 48 h after the fungi accessed a ^15^N‐labelled N source. Selected images (a–c) showing representative examples of external hyphae at high, medium and low isotopic enrichments. (d) Breakpoint linear regression analysis showing ^13^C and ^15^N enrichment (at% excess, APE) within regions of interest (ROIs) of all external hyphae in the cross‐section of the root tip; the open triangles and the dashed line represent the cell walls; the closed circles and the solid line represent the lumen; (d) also shows the positions of both the cell walls (CW) and lumen (L) of the selected hyphae in the regression analyses: a, relatively low ^15^N and almost no ^13^C enrichment, below breakpoint (BP); b, medium enrichment, around the BP; and c, high enrichment, above the BP. The line plots on the right side of each image series show isotopic enrichment profiles across each of the three selected hyphae (solid red line: at%^13^C; dashed blue line: at%^15^N) obtained from line‐scan analysis at the positions indicated by the white lines in panels (a)‐I, (b)‐I and (c)‐I. The colour scales at the bottom of the ^13^C and ^15^N NanoSIMS images range from blue (natural abundance) to red (high isotopic enrichment). Bars: (a–c) 1 µm.

We observed two typical patterns of ^13^C and ^15^N spatial distribution in cross‐sectioned external hyphae within all NanoSIMS images (indicated in Figs 5, 6), three examples of which are outlined in more detail in Fig. 8. These two patterns can be summarised as follows: first, when hyphae had a relatively low overall enrichment of ^13^C (i.e. they were located below the regression breakpoint, Fig. 8a,d), ^15^N was mainly located in the middle of the hyphae (Fig. 8a, further examples are indicated by white arrows in Figs 5, 6), while ^13^C enrichment was observed in the outer parts of cross‐sectioned hyphae (i.e. not spatially correlated with ^15^N at that scale); second, at relatively high ^13^C (and ^15^N) enrichments (i.e. above the regression breakpoint, Fig. 8c,d) ^15^N and ^13^C were present all over the cross‐section, but their enrichments peaked in the outer ring of the hyphae, where they were strongly spatially correlated (Fig. 8c,d and red arrows in Figs 5, 6).

## Discussion

We conducted a ^13^C and ^15^N double labelling experiment to trace the path of recently assimilated plant C and fungus‐delivered N through an ectomycorrhizal system. If plants allocate more C to N‐delivering fungal partners, we expect ^13^C and ^15^N to be correlated at a given spatial scale – that is, across root segments, root tips or cells within a root tip. While we observed no link between ^13^C and ^15^N at the root system scale, we found a surprisingly strong spatial correlation within the mycorrhizal tissue of a single root tip.

Our plants received a significant amount of ^15^N‐enriched N within 48 h via mycorrhizal fungi associated with half of their root system. On average, 20.2 µg of excess ^15^N was found in each plant, corresponding to *c*. 7% of the ^15^N added to the litter compartments (Gorka *et al*., 2019). The majority of this N was found in the root system half connected to the ^15^N‐receiving litter compartment (Fig. 2a). Despite the differences in additional N uptake between the two root system halves, photoassimilated C was equally allocated to both (Fig. 2b), indicating that plants did not allocate more C to parts of the root system which had access to greater quantities of N. We did, however, observe a correlation between at%^13^C and at%^15^N across individual cm‐sized root segments in each of the root system halves (Fig. 2c). This correlation, though, was far more significant in root segments of the N‐unamended side, where ^15^N could only arrive by internal redistribution within the plant (Fig. 2c). This indicates that ^13^C was more likely to be attracted to places that the newly obtained ^15^N had been relocated to – so that they could be used together for growth or maintenance, for example – rather than to the places colonized by N‐delivering fungal partners. These results are in contrast to the findings of Bogar *et al*. (2019) who showed, also in a split‐root experiment, that *Pinus* seedlings directed recent photosynthates preferentially to roots and mycorrhizas that had access to an additional nitrogen source. The different outcomes may be the result of the different temporal scales of the experiments, as Bogar *et al*. (2019) supplied additional nitrogen over a time period of four months, while we only provided a one‐time addition shortly before ^13^CO_2_ labelling. It could, however, also be attributed to differences in C allocation strategies in specific host‐symbiont pairings.

The enrichment of ^13^C and ^15^N across mycorrhizal root tips was much more heterogenous than that observed in the root segments. Although there was no significant correlation, it seems that root tips with a high ^15^N enrichment were also always highly enriched in ^13^C, whereas root tips with a high ^13^C enrichment did not necessarily show a high ^15^N enrichment (Fig. 2d). This could mean that plants not only gave more C to N‐delivering fungi, but also to fungi bringing other benefits/nutrients, which we did not measure. In addition, as the roots of our plants were colonized by different fungal species, it could also be the result of fungal‐species specific plant C allocation (Pena & Polle, 2013). Interestingly, there were no root tips with high ^15^N but low ^13^C, demonstrating that plants may indeed need to invest recently photoassimilated C into the acquisition of N from fungi.

Nanoscale secondary ion mass spectrometry imaging revealed novel insights into the spatial distribution of recent photosynthates and fungus‐delivered N at the cellular scale in an ectomycorrhizal root tip. Many of the external hyphae in our root cross‐section were significantly, and highly, labelled in ^15^N, indicating that they had been rapidly taking up ^15^N from the litter compartment. The fact that the vascular bundle of the root was also highly enriched in ^15^N (up to 5 APE), indicates that the fungi passed on a considerable share of the N they took up to the plants. The high heterogeneity in ^15^N enrichment across individual external fungal hyphae (ranging from 0.48 to 21.30 at%^15^N enrichment) could be the result of hyphal ends growing into different areas of soil and litter compartments, allowing some hyphae to access more ^15^N than others. Surprisingly, the heterogenous spatial distribution of ^15^N was matched by a similarly heterogenous distribution of ^13^C (Figs 5, 6). In particular, external fungal hyphae that were extraordinarily highly enriched in ^15^N were also extraordinarily highly enriched in ^13^C, with values often almost as high as those of the plant's phloem (up to 5.11 at%^13^C, Table 1).

The high heterogeneity of ^13^C distribution across the fungal mantle and external hyphae, and its strong link to the ^15^N distribution, is astonishing. To the best of our knowledge, our study is the first, or one of the first, to visualize the distribution of recent photosynthates and fungus‐delivered N at the scale of individual cells in the ectomycorrhizal tissue. Bücking & Heyser (2001) investigated the small‐scale distribution of ^14^C‐labelled photosynthates and ^33^P‐labelled P in longitudinal sections of ectomycorrhizal root tips of poplar seedlings using microautoradiography. In contrast to our results, they reported a homogenous distribution of photosynthates across the fungal sheath, which could perhaps be ascribed to a longer incubation period of 5 d after their seedlings were exposed to a ^14^CO_2_ pulse, or a lower spatial resolution.

Relative enrichments of ^13^C and ^15^N were significantly spatially correlated across the whole root tip cross‐section (Figs 7, S5), indicating that N recently taken up by mycorrhizal fungi is coupled to recently plant‐assimilated C within the mycorrhizal tissue. When interpreting this pattern, we have to bear in mind the fact that our NanoSIMS analysis represents a snapshot in time shortly (*c*. 24 h) after ^13^C entered the plant via photosynthesis and ^15^N was taken up by mycorrhizal fungi. The ^13^C and ^15^N distribution we observe in the plant and fungal cells could be the result of a variety of physiological processes, including solute transfer, growth, and the build‐up of storage compounds.

The strong co‐location of ^13^C and ^15^N in plant and fungal cells could in principle reflect growth processes which utilize both plant‐assimilated C and N from fungal uptake. For the emanating (‘external’) fungal hyphae in our cross‐sections however, we think that this would be an unlikely scenario. Fungal hyphae grow in a polarized manner, by apical extension exclusively at the hyphal tip (requiring a specialised enzymatic machinery and a long‐range tip‐ward transport of secretory vesicles; Lew, 2011; Riquelme, 2013; Steinberg *et al*., 2018). These growing tips of ectomycorrhizal hyphae emanating from roots are located downstream in the soil, or in our case – as indicated by high ^15^N enrichments – most likely in the litter compartments. The growth of new hyphal tissue in emanating hyphae within a few µm around the root can only occur if new hyphal branches form there. Given that we captured around 200 individual emanating hyphae in our NanoSIMS images, we think – even if some new hyphal branches may have formed in the last 24 h – that a large proportion of them likely represent hyphae that were already established, and thus would grow only at their remote tips. We therefore think that growth processes are insufficient to explain the strong correlation between at%^13^C and at%^15^N we observed across external hyphae.

The observation that ^15^N enrichment was on average higher in fungal cells than plant cells (Fig. S4) is probably due to the fact that fungi had access to the ^15^N first. However, it could also reflect an accumulation of ^15^N in fungal tissue which is not transferred to the host plant. It has been hypothesised that ectomycorrhizal fungi hoard N in N‐limited situations (Corrêa *et al*., 2012; Näsholm *et al*., 2013). Although fungal storage of N in the mantle is in principle possible, we did not find strong signs of accumulation of N in fungal tissues. In fact, strong correlations only occurred between the *relative* isotopic enrichments (i.e. in at% or APE, Figs 7, S5) in fungal tissues, but there was no systematic increase in total N with relative enrichments of ^15^N (or ^13^C) (Fig. S5). This indicates that the freshly taken up ^15^N did not accumulate on top of the existing N, but rather replaced it. For example, a relative enrichment of around 20 at%^15^N in external hyphae without a corresponding increase in total N means that one fifth of their N has been replaced by the new labelled source (which had a relative enrichment of 99 at%^15^N) within 48 h. This points towards a rapid turnover of N in external hyphae, which we might expect to occur under the active transport of N. We therefore think that it is more likely that the relative ^15^N enrichment of external hyphae reflects N in active transport processes towards the plant–fungal interface, rather than growth or local storage of N.

Characteristics and qualities of at%^13^C : at%^15^N regressions (i.e. slope, *R* ^2^) substantially differed between ectomycorrhizal tissue types (Table 2; Figs 7, S5). For example, the slope of the regression (which can be interpreted as the increase in ^15^N per increase in ^13^C) was much steeper in the lumen compared to the cell walls across all fungal tissues (Table 2). These differences indicate that the spatial distribution of recently photoassimilated C and fungus‐delivered N was governed by different mechanisms in the different tissue types.

Interestingly, we found biphasic trends of correlations between ^13^C and ^15^N in all tissues forming the mycorrhizal root tip (Fig. 7). In all cases, a strong linear relationship between ^15^N and ^13^C enrichment occurred up to a certain threshold, from which further increases in ^13^C enrichment were accompanied by only marginal enrichments in ^15^N. Different explanations are possible for such a pattern. One could be that substantially different mechanisms are driving the pattern below and above the breakpoint; for example, it could be caused by transport processes below the threshold but reflect growth of new tissue above it. Alternatively, certain ‘threshold’ amounts of labile N could trigger changes in cell metabolism, signalling or transporter activity, which may increase the C sink strength of the tissue. Finally, under the assumption that the observed spatial patterns reflect transport processes, the observed ‘threshold’ relationship could indicate that obtaining larger quantities of N requires a disproportionately higher C investment compared to smaller quantities (Kummel & Salant, 2006). This could be because small quantities may be readily accessible by the existing fungal hyphal network, whereas larger ones may require additional C‐costly hyphal growth and foraging (e.g. fan formation).

Interestingly, we identified contrasting spatial patterns of ^13^C and ^15^N distribution in cross‐sectioned external hyphae at low and high isotopic enrichments (Fig. 8). These patterns are difficult to interpret as not much is known about the bi‐directional transfer of C and N in ectomycorrhizal hyphae. Solutes can be transported in fungal mycelia by a variety of mechanisms, such as diffusion, mass flow, motor‐driven vesicular transport or cytoplasmic streaming (Cairney, 2005; Lew, 2011). Nitrogen is known to be transported in the form of amino acids through fungal hyphae (Cruz *et al*., 2007; Chalot & Plassard, 2011; Koide *et al*., 2014). It has recently been proposed that this happens within a dynamic network of vacuoles linked by tubules through the ectomycorrhizal mycelia (Ashford & Allaway, 2002; Darrah *et al*., 2006; Fricker *et al*., 2018; Nehls & Plassard, 2018). Vacuole transport, however, is based on diffusion, and is therefore relatively slow (Fricker *et al*., 2018). The fact that the added N‐compounds in our system have travelled a distance of *c*. 8 cm within 48 h indicate that nondiffusion‐based transport mechanisms, such as mass‐flow or cytoplasmic streaming, were also involved. In both mass‐flow and cytoplasmic streaming, nutrients are channelled through the septal pores in the middle of the hyphae, which may explain why N appears to be highest in the center of the cross‐sectioned hyphae in the ‘relatively low isotopic enrichment’ type (Fig. 8a, white arrows in Figs 5, 6). The observed colocation of high ^13^C and ^15^N enrichments in the outer ring of ‘relatively high isotopic enrichment’ hyphae (Fig. 8c, red arrows in Figs 5, 6), on the other hand, could represent temporary storage of ^13^C and ^15^N co‐labelled amino acids in the dynamic tubular vacuole network, which has been suggested to be linked to hyphal walls (Ashford & Allaway, 2002; Darrah *et al*., 2006), and to serve as temporary storage for N in the form of amino acids upon their export to the plant as NH_4_ (Nehls & Plassard, 2018). The co‐location of high ^13^C and ^15^N enrichments in the outer ring, could, however, alternatively also resemble growth of newly branched hyphal tips, with the obvious limitations discussed in the paragraph on growth above. We also cannot rule out the possibility that the high isotopic enrichments in the outer ring could be a result of bacteria colonizing the hyphal surface and feeding on hyphal exudates (Gorka *et al*., 2019).

We analysed twenty 70 × 70 µm fields of view using NanoSIMS to visualize the isotopic distribution of C and N in a complete cross‐section of an ectomycorrhizal root tip. While our results provide novel insights into the spatial distribution of recent photosynthates and fungus‐delivered N in the ectomycorrhizal tissue, they nonetheless only allow conclusions to be drawn for one specific ectomycorrhizal association. Further studies are needed to understand whether different ectomycorrhizal associations exhibit different C and N distribution patterns, and to assess their temporal dynamics. Still, our results demonstrate the power of combining stable isotope tracing with NanoSIMS to investigate C and N exchange at the cellular scale in the mycorrhizal symbiosis, and highlight the fact that processes governing this exchange may operate even at the small‐scale of individual emanating fungal hyphae.

## Author contributions

CK conceived the idea for the study, and developed the experimental design together with AR and DW. WM, SG, JW, VM, MD and RG carried out the experimental work under the supervision of CK, AR and DW. RG and MD did preliminary tests. MD carried out the sequencing analysis of the root tip sample. PC, M Wagner, M Weidinger, SR and AS contributed to the development of the method of visualizing labile C and N compounds in ectomycorrhizal tissue using NanoSIMS. WM carried out NanoSIMS sample preparation with support from SR. AS performed the NanoSIMS measurements. WM did the image processing and data analysis and wrote the first draft of the paper, supported by CK. All co‐authors contributed to the paper revision, and CK wrote the final version of the paper.

## Supporting information


**Fig. S1** Macro photography of an ectomycorrhizal root tip of beech.
**Fig. S2** ^12^C^14^N^−^ secondary ion signal intensity distribution images recorded in the first analysis run.
**Fig. S3** ^12^C^14^N^−^ secondary ion signal intensity distribution and inferred at%^15^N distribution of consecutive fields of view without additional pre‐sputtering.
**Fig. S4** Stable isotope enrichment of ^13^C and ^15^N in plant and fungal tissues measured on a cross‐section of an ectomycorrhizal root tip of beech (*Fagus sylvatica*) determined via NanoSIMS.
**Fig. S5** Correlations between relative abundances of ^13^C and ^15^N (at%), and total N in regions of interest (ROIs) of a cross‐section of a mycorrhizal root.
**Fig. S6** Nanoscale secondary‐ion mass (NanoSIMS) visualization of the ^13^C label distribution in a beech ectomycorrhizal root tip cross‐section.
**Fig. S7** NanoSIMS visualization of the ^15^N label distribution in a beech ectomycorrhizal root tip cross‐section.
**Fig. S8** Colour‐blind friendly NanoSIMS visualization of the ^13^C label distribution in a beech ectomycorrhizal root tip cross‐section.
**Fig. S9** Colour‐blind friendly NanoSIMS visualization of the ^15^N label distribution in a beech ectomycorrhizal root tip cross‐section.
**Fig. S10** NanoSIMS total CN^−^ secondary ion signal intensity distribution image of a beech ectomycorrhizal root tip cross‐section (*Fagus sylvatica* and *Thelephora* fungi), visualizing the cellular structure of the sample.
**Methods S1** Additional methodological details of the NanoSIMS analysis.
**Notes S1** Potential bias of ^15^N measurements due to N_2_ adsorption during consecutive NanoSIMS analyses of multiple fields of view on one sample.
**Table S1** Regression analysis of ^13^C vs ^15^N isotope enrichment (at% excess, APE) in distinct tissue types of an ectomycorrhizal root tip.
Please note: Wiley Blackwell are not responsible for the content or functionality of any Supporting Information supplied by the authors. Any queries (other than missing material) should be directed to the New Phytologist Central Office.

## Data Availability

The data that support the findings of this study are openly available on Zenodo at 10.5281/zenodo.5035482 (Mayerhofer *et al*., 2021). The sequence data were deposited in the National Center for Biotechnology Information (NCBI) Short Read Archive under study accession no. PRJNA606050.
