# Supplementary material for: Recently photoassimilated carbon and fungus‐delivered nitrogen are spatially correlated in the ectomycorrhizal tissue of Fagus sylvatica
Source: New Phytol. 2021 Aug 6;232(6):2457–74. doi: 10.1111/nph.17591 (PMC9291818; doi:10.1111/nph.17591)
Supplement: Supplementary file 1 — Fig. S1 Macro photography of an ectomycorrhizal root tip of beech. Fig. S2 12C14N− secondary ion signal intensity distribution images recorded in the first analysis run. Fig. S3 12C14N− secondary ion signal intensity distribution and inferred at%15N distribution of consecutive fields of view without additional pre‐sputtering. Fig. S4 Stable isotope enrichment of 13C and 15N in plant and fungal tissues measured on a cross‐section of an ectomycorrhizal root tip of beech (Fagus sylvatica) determined via NanoSIMS. Fig. S5 Correlations between relative abundances of 13C and 15N (at%), and total N in regions of interest (ROIs) of a cross‐section of a mycorrhizal root. Fig. S6 Nanoscale secondary‐ion mass (NanoSIMS) visualization of the 13C label distribution in a beech ectomycorrhizal root tip cross‐section. Fig. S7 NanoSIMS visualization of the 15N label distribution in a beech ectomycorrhizal root tip cross‐section. Fig. S8 Colour‐blind friendly NanoSIMS visualization of the 13C label distribution in a beech ectomycorrhizal root tip cross‐section. Fig. S9 Colour‐blind friendly NanoSIMS visualization of the 15N label distribution in a beech ectomycorrhizal root tip cross‐section. Fig. S10 NanoSIMS total CN− secondary ion signal intensity distribution image of a beech ectomycorrhizal root tip cross‐section (Fagus sylvatica and Thelephora fungi), visualizing the cellular structure of the sample. Methods S1 Additional methodological details of the NanoSIMS analysis. Notes S1 Potential bias of 15N measurements due to N2 adsorption during consecutive NanoSIMS analyses of multiple fields of view on one sample. Table S1 Regression analysis of 13C vs 15N isotope enrichment (at% excess, APE) in distinct tissue types of an ectomycorrhizal root tip. Please note: Wiley Blackwell are not responsible for the content or functionality of any Supporting Information supplied by the authors. Any queries (other than missing material) should be directed to the New Phytologist Central Office. [file NPH-232-2457-s001.pdf]

***New Phytologist* Supporting Information**

Article title: Recently photoassimilated Carbon and fungal-delivered Nitrogen are spatially correlated at the cellular scale in the ectomycorrhizal tissue of Fagus sylvatica

Authors: Werner Mayerhofer, Arno Schintlmeister, Marlies Dietrich, Stefan Gorka, Julia Wiesenbauer, Victoria Martin, Raphael Gabriel, Siegfried Reipert, Marieluise Weidinger, Peta Clode, Michael Wagner, Dagmar Woebken, Andreas Richter, Christina Kaiser

Article acceptance date: 01 June 2021

The following Supporting Information is available for this article:

Fig. S1 Macro Photography of an ectomycorrhizal root tip of beech. Scale bar, 0.5 mm.

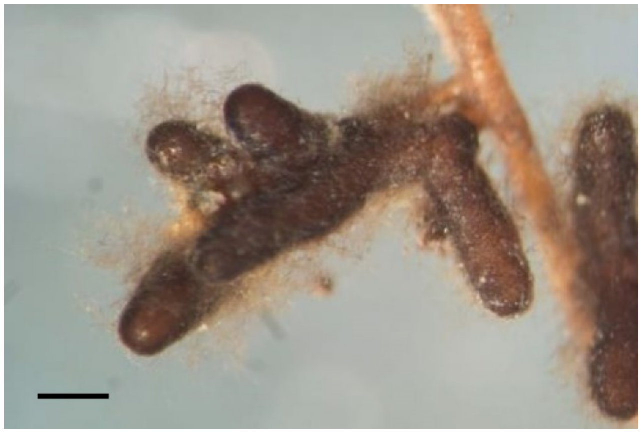

Fig. S2 $^{12}C^{14}N^{-}$ secondary ion signal intensity distribution images recorded in the first analysis run. The number at the bottom of each panel refers to the chronological order of acquisition (sample stage movement). The grid pattern in the first image originates from spectrometer tuning, performed at a raster scheme of 64 x 64 pixels. The rectangular regions exhibiting low signal intensity indicate sample areas exposed to primary ion bombardment during acquisition of a preceding image. The size of each individual image is 55 x 55 µm.

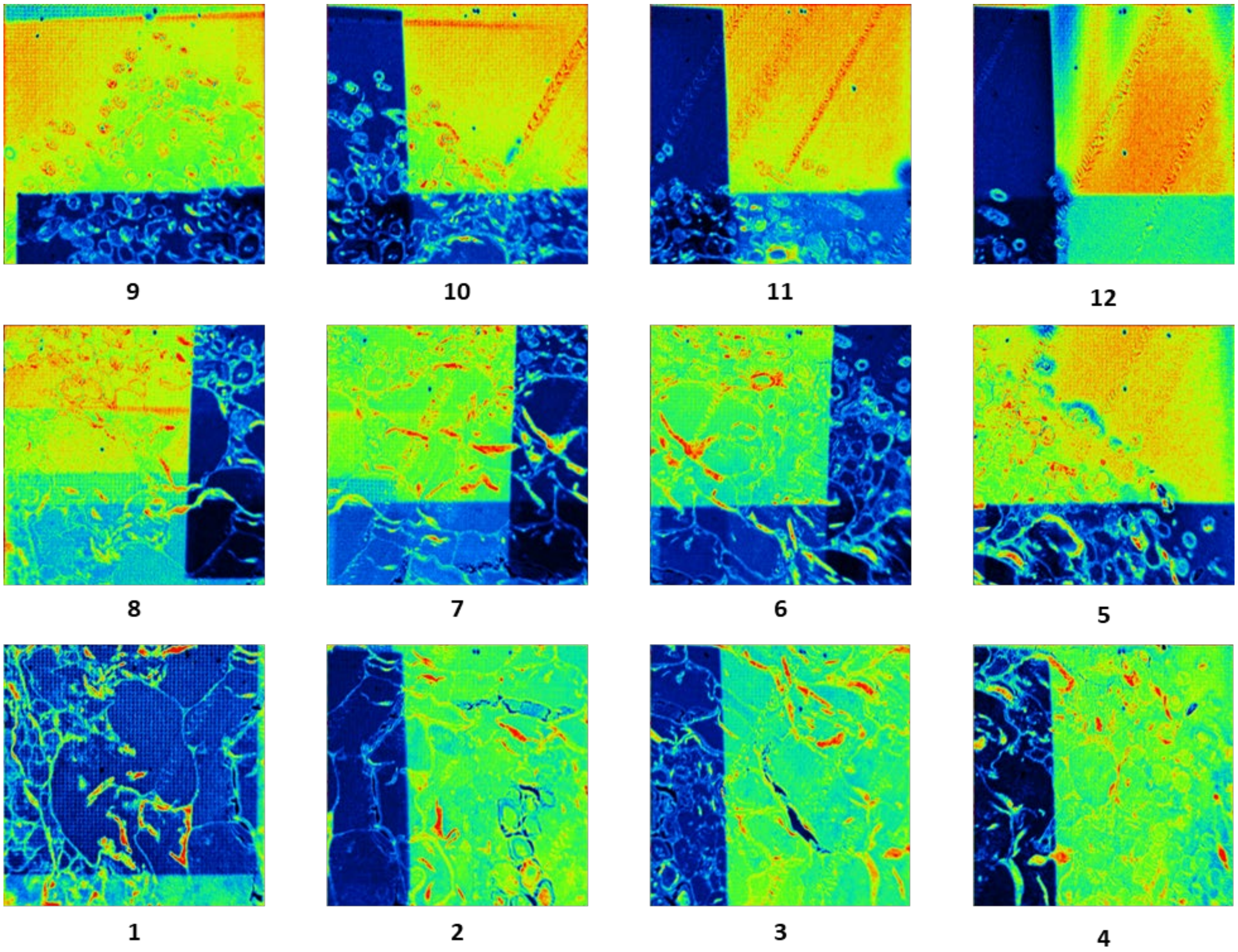

Fig. S3 $^{12}C^{14}N^-$ secondary ion signal intensity distribution and inferred ^{15}N at% distribution of consecutive fields of view without additional pre-sputtering. Left: $^{12}C^{14}N^-$ secondary ion signal intensity distribution images number 7,8,9 and 10 recorded in the first analysis run (see Fig. S2 for the complete array). Right: ^{15}N at% distribution as inferred from per-pixel calculation of $^{12}C^{14}N^-/(^{12}C^{14}N^- + {}^{12}C^{15}N^-)$ signal intensity ratios. White rectangles indicate areas that were exposed to primary ion bombardment during acquisition of a preceding image. Note the bias in the ^{15}N content within these areas resulting from adsorption of nitrogen containing molecules (mainly N_2) with natural isotopic abundance in the residual gas of the analysis chamber. The size of each individual image is 55 x 55 µm.

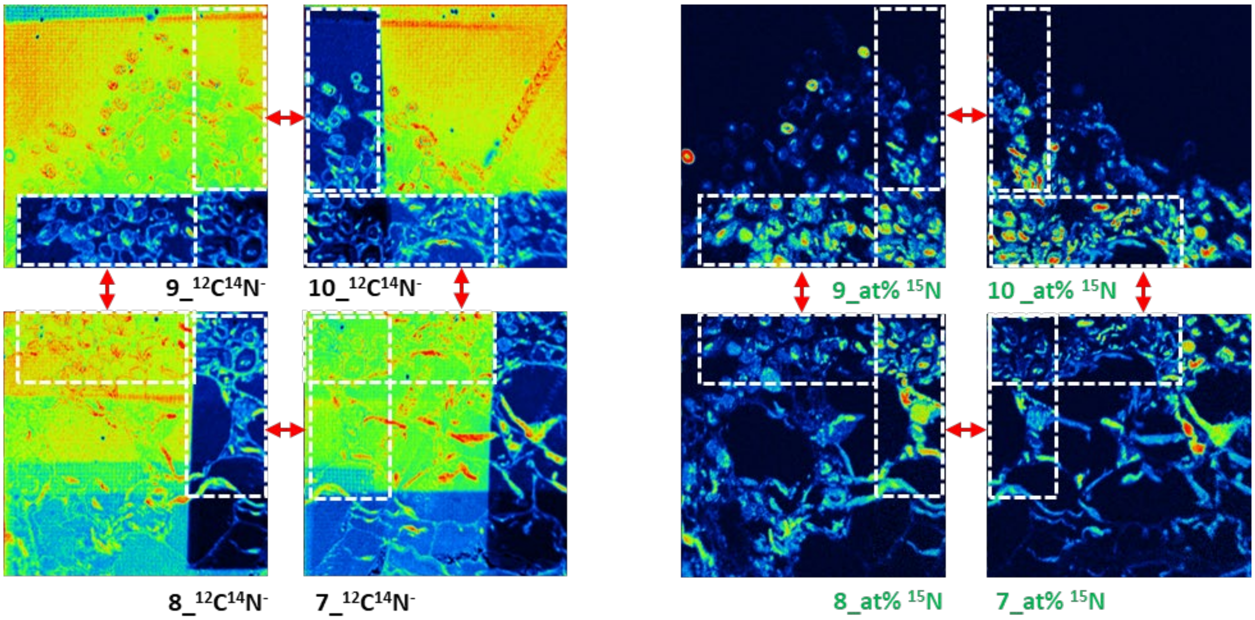

Fig. S4 Stable isotope enrichment of ^{13}C and ^{15}N in plant and fungal tissue measured on a cross section of an ectomycorrhizal root tip of beech (*Fagus sylvatica*) determined via NanoSIMS. Histological predefined tissue categories from outside (left) to inside (right): fungi: HE – hyphae extended, HM – hyphae mantle, HN – hyphae Hartig net; plant: PC – plant cortex, E – endodermis, VT – vascular tissue; cell walls: open circles (○), lumen: closed circles (●).Isotope enrichment is presented as atom% excess (APE), i.e. labelled sample minus unlabelled control. Displayed are the means of n ROIs for each tissue category (n=24-424, see Table 1), with error bars referring to one standard error (SE). Significant differences are depicted as letters calculated by a generalized linear model (GLM) with different link functions, based on data distribution of enrichment: ^{13}C – Gamma log link and ^{15}N – Gauss log link with subsequent application of a Tukey Post-hoc test.

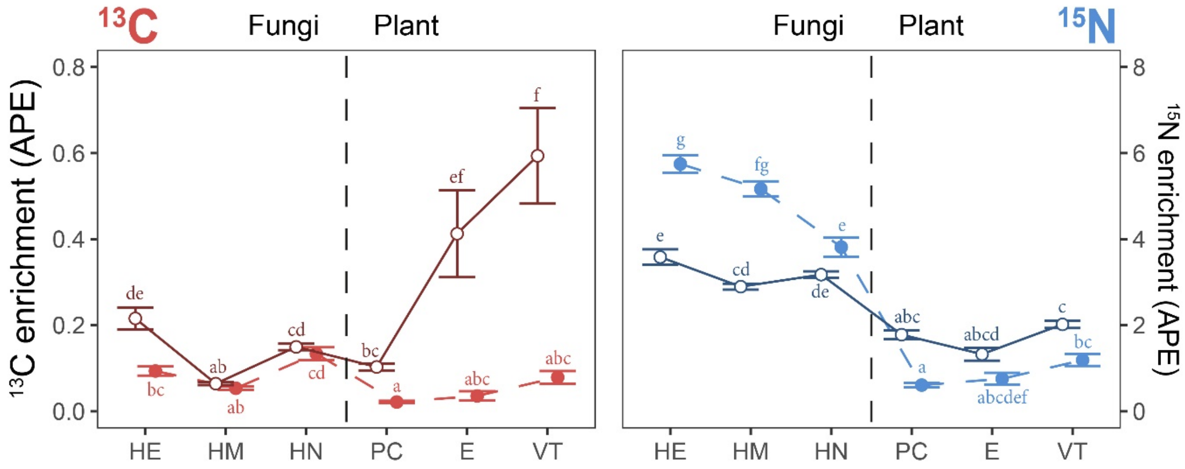

Fig. S5 Correlations between relative abundances of ^{13}C and ^{15}N (atom%), and total N in regions of interest (ROIs) of a cross section of a mycorrhizal root. Each dot represents one ROI defined in NanoSIMS images based on tissue type (Extended Hyphae, Hartig Net, Hyphal mantle, Plant cortex cells, plant endodermis, plant vascular bundle) and cell part (lumen, cell wall). at% ^{13}C values were inferred from the C_2^- secondary ion signal intensity distribution images via per-pixel calculation of $^{13}C^{12}C$-/(2·$^{12}C^{12}C$-+$^{13}C^{12}C$-) intensity ratios. at% ^{15}N values were inferred from the ^{12}CN- secondary ion signal intensity maps via per-pixel calculation of $^{12}C^{15}N$-/($^{12}C^{15}N$-+$^{12}C^{14}N$-) intensity ratios. Total N is calculated as the sum of $^{12}C^{14}N$- and $^{12}C^{15}N$- signal intensities.

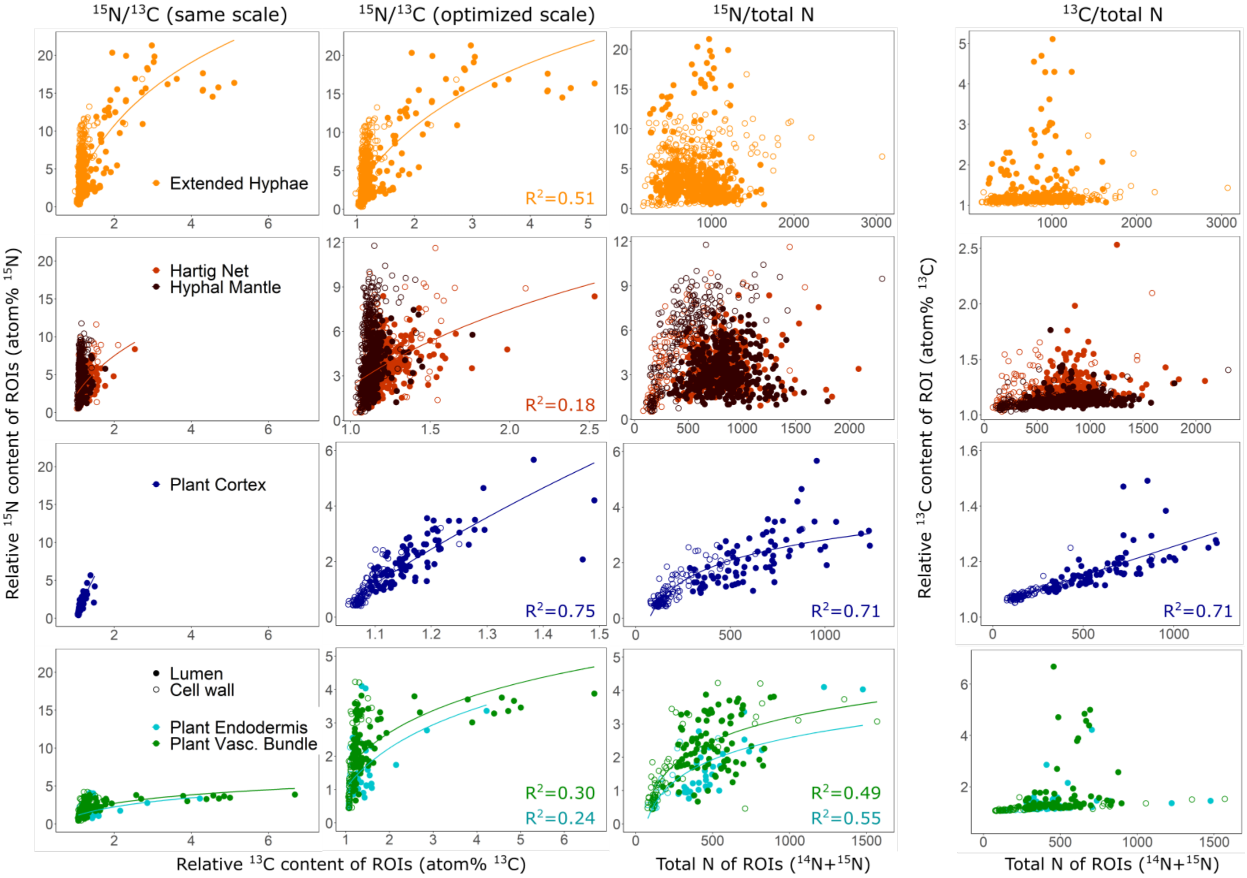

Fig. S6 NanoSIMS visualization of the ^{13}C label distribution in a beech ectomycorrhizal root tip cross section. The ^{13}C label content is displayed as atom%^{13}C, indicating isotopically labelled recent photosynthates (24h after labelling) in a cross section of an ectomycorrhizal root tip of beech (*Fagus sylvatica*) and *Thelephora* fungi. The picture consists of 16 individual images (each 50x50 µm), assembled as a mosaic. The colour scale at the bottom ranges from the natural abundance value (determined on an unlabelled control) to 3 at% ^{13}C. Secondary ion ($^{12}C^{13}C^-$ and $^{12}C_2^-$) signal intensity thresholds were set to 20 counts per (sec x pixel). Black areas refer to regions in which the intensities were below that threshold, impeding accurate determination of the ^{13}C content on the per-pixel level due to low counting statistics. Scale bar = 50 µm.

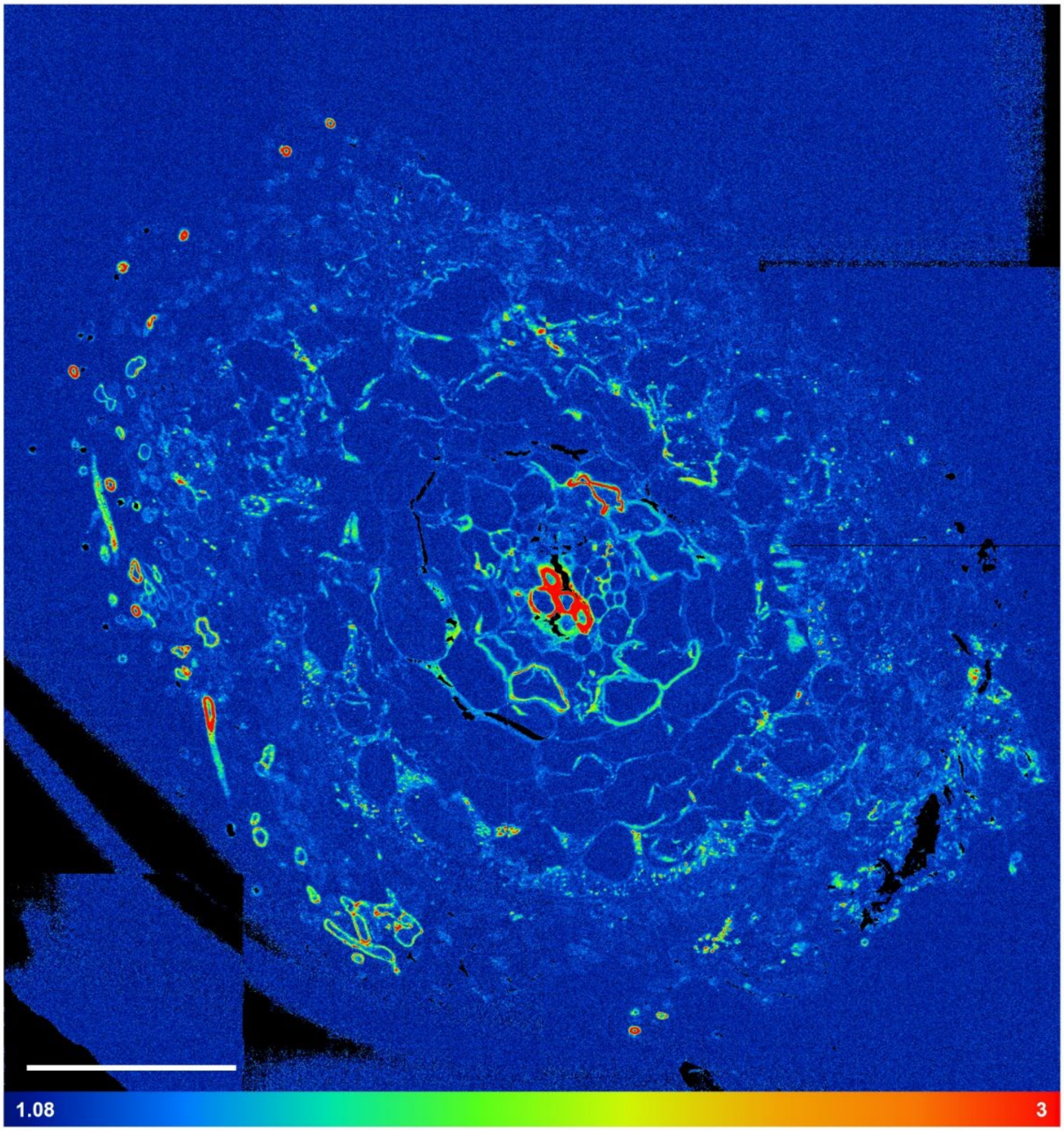

Fig. S7 NanoSIMS visualization of the ^{15}N label distribution in a beech ectomycorrhizal root tip cross section. ^{15}N label content is displayed as atom%^{15}N, indicating isotopically labelled fungal and plant metabolites on a cross section of an ectomycorrhizal root tip of beech (*Fagus sylvatica*) and *Thelephora* fungi. The picture consists of 16 individual images (each 50x50 µm), assembled as a mosaic. The colour scale at the bottom ranges from the natural abundance value (determined on an unlabelled control) to 20 at% ^{15}N. Secondary ion ($^{12}C^{14}N^-$ and $^{12}C^{15}N^-$) signal intensity thresholds were set to 7 counts per (sec x pixel) in order to exclude areas in which low counting statistics impede accurate determination of the ^{15}N content on the per-pixel level. Scale bar = 50 µm

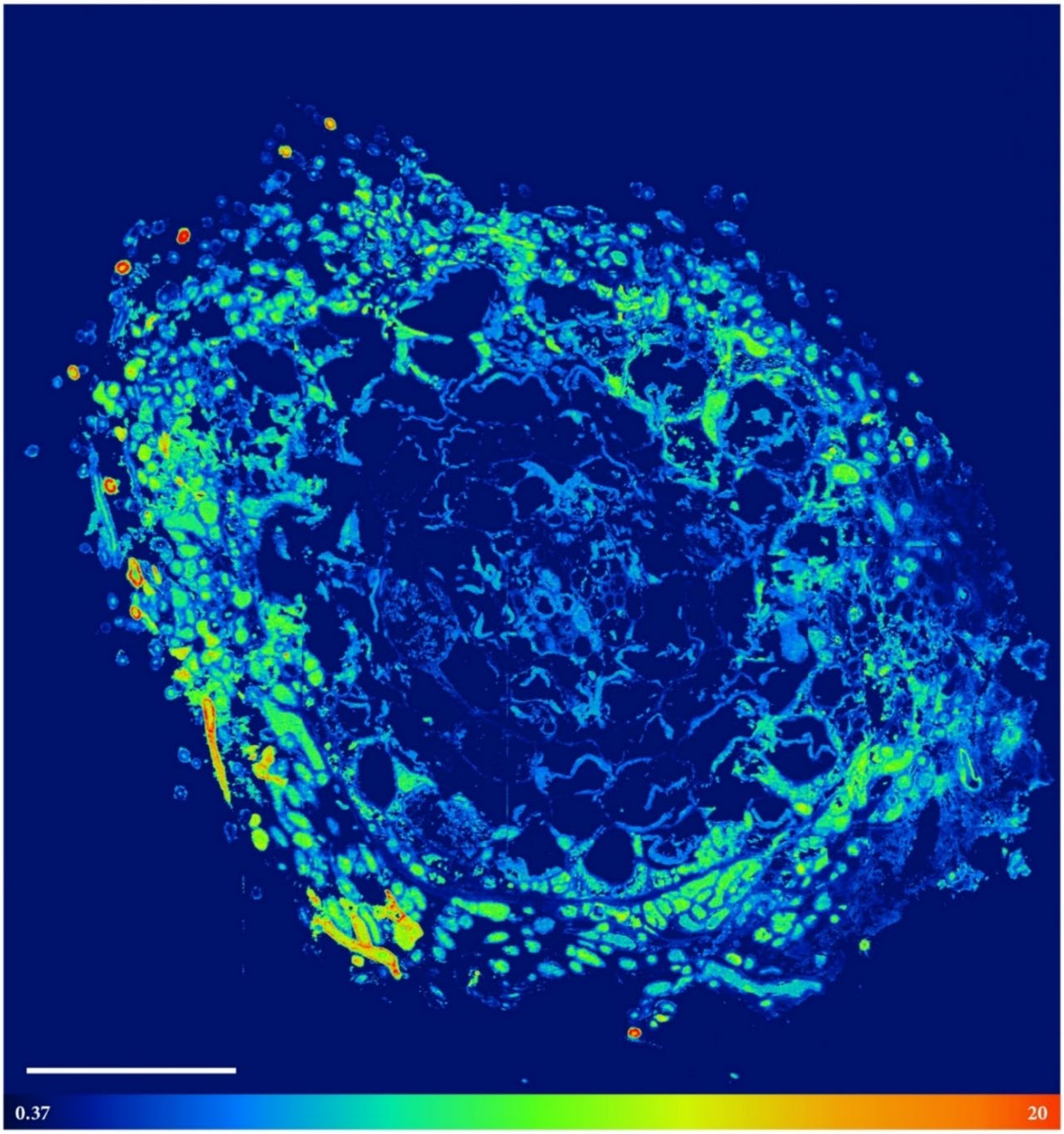

Fig. S8 Colour blind friendly NanoSIMS visualization of the ^{13}C label distribution in a beech ectomycorrhizal root tip cross section. Displayed is the same NanoSIMS image as presented in Fig. S6, utilizing a blue-to-yellow color scale for visualization of the ^{13}C label distribution, given in atom% ^{13}C. Secondary ion ($^{12}C^{13}C^-$ and $^{12}C_2^-$) signal intensity thresholds were set to 20 counts per (sec x pixel). Dark blue areas refer to regions in which the intensities are below that threshold, impeding accurate determination of the ^{13}C content on the per-pixel level due to inferior counting statistics. Scale bar = 50 µm.

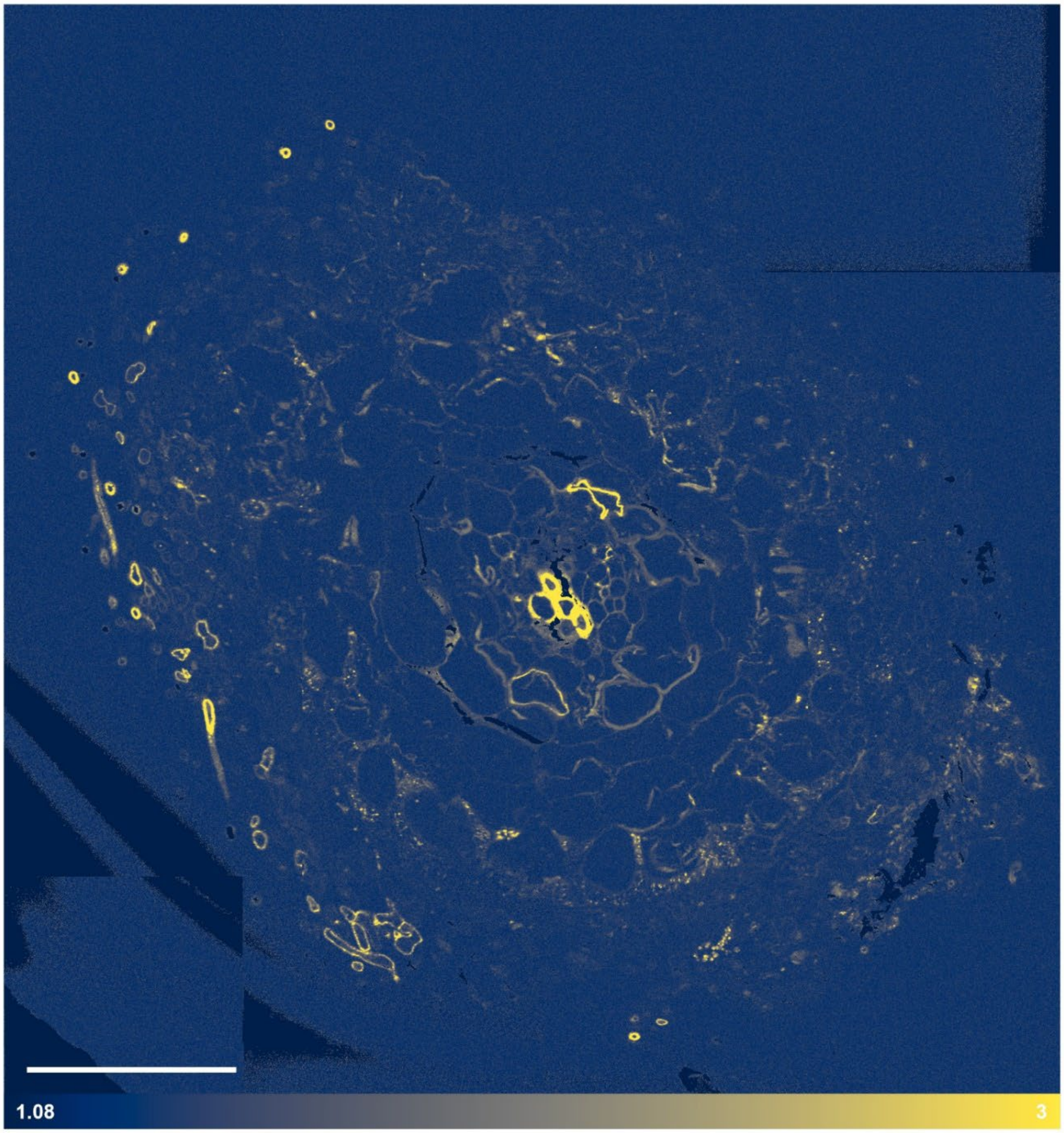

Fig. S9 Colour blind friendly NanoSIMS visualization of the ^{15}N label distribution in a beech ectomycorrhizal root tip cross section.. Displayed is the same NanoSIMS image as presented in Fig. S7, utilizing a blue-to-yellow color scale for colour blind friendly visualization of the ^{15}N label distribution, given in atom% ^{15}N. Secondary ion ($^{12}C^{14}N^-$ and $^{12}C^{15}N^-$) signal intensity thresholds were set to 7 counts per (sec x pixel). Dark blue areas refer to regions in which the intensities are below that threshold in order to exclude areas in which low counting statistics impede accurate determination of the ^{15}N content on the per-pixel level. Scale bar = 50 µm.

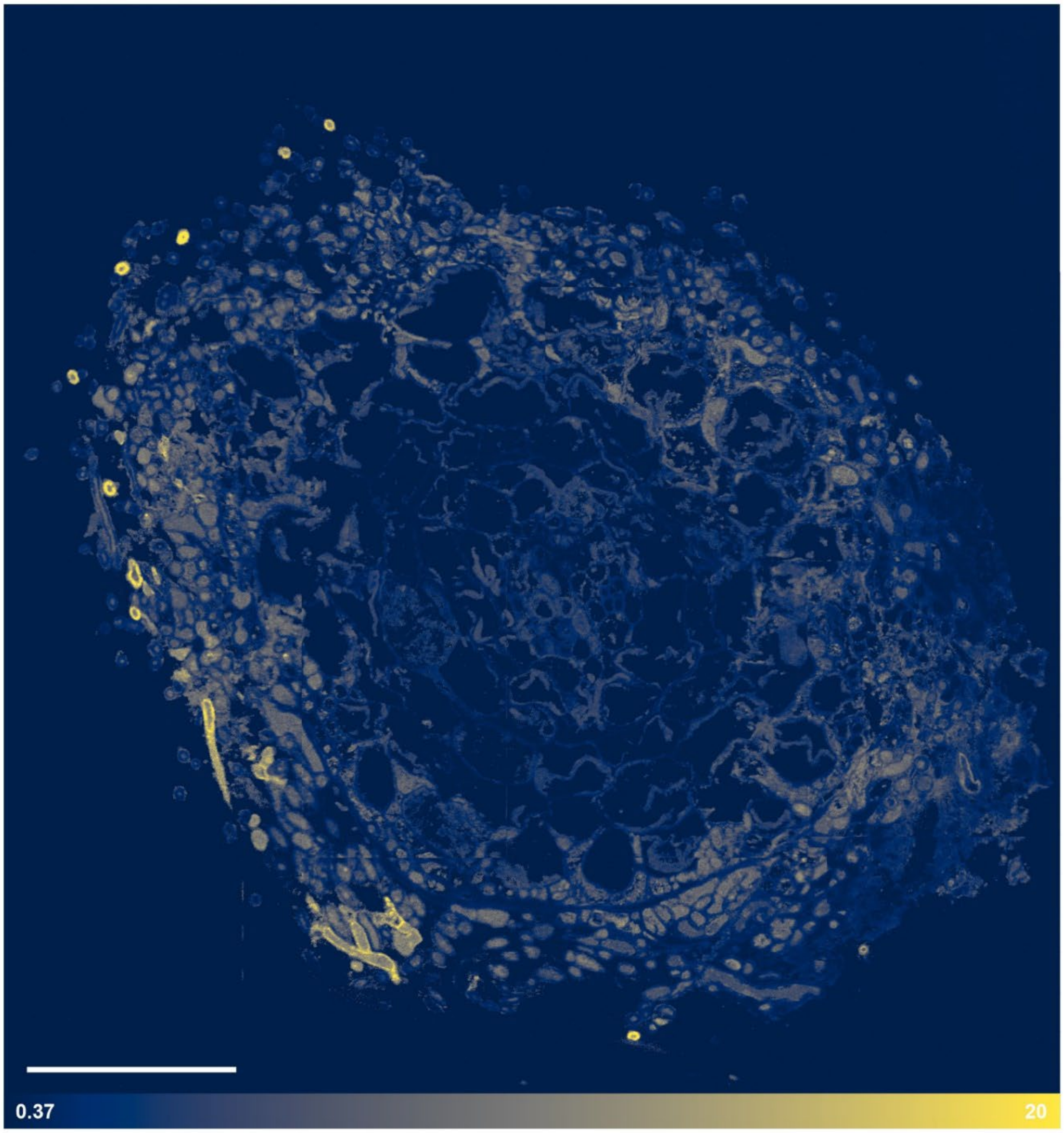

Fig. S10 NanoSIMS total CN^- secondary ion signal intensity distribution image of a beech ectomycorrhizal root tip cross section (Fagus sylvatica and Thelephora fungi), visualizing the cellular structure of the sample. The signal intensity is displayed on a greyscale ranging from black (low signal intensity) to white (high signal intensity). Histologically distinct regions within the tissue refer to: HE – extended hyphae, HM – hyphae mantle, HN – Hartig net, PC – plant cortex, E – endodermis, VT – vascular tissue. Letters a-c refer to the regions of the line-scan-analysis (Fig. 8). Scale bar = 50 µm

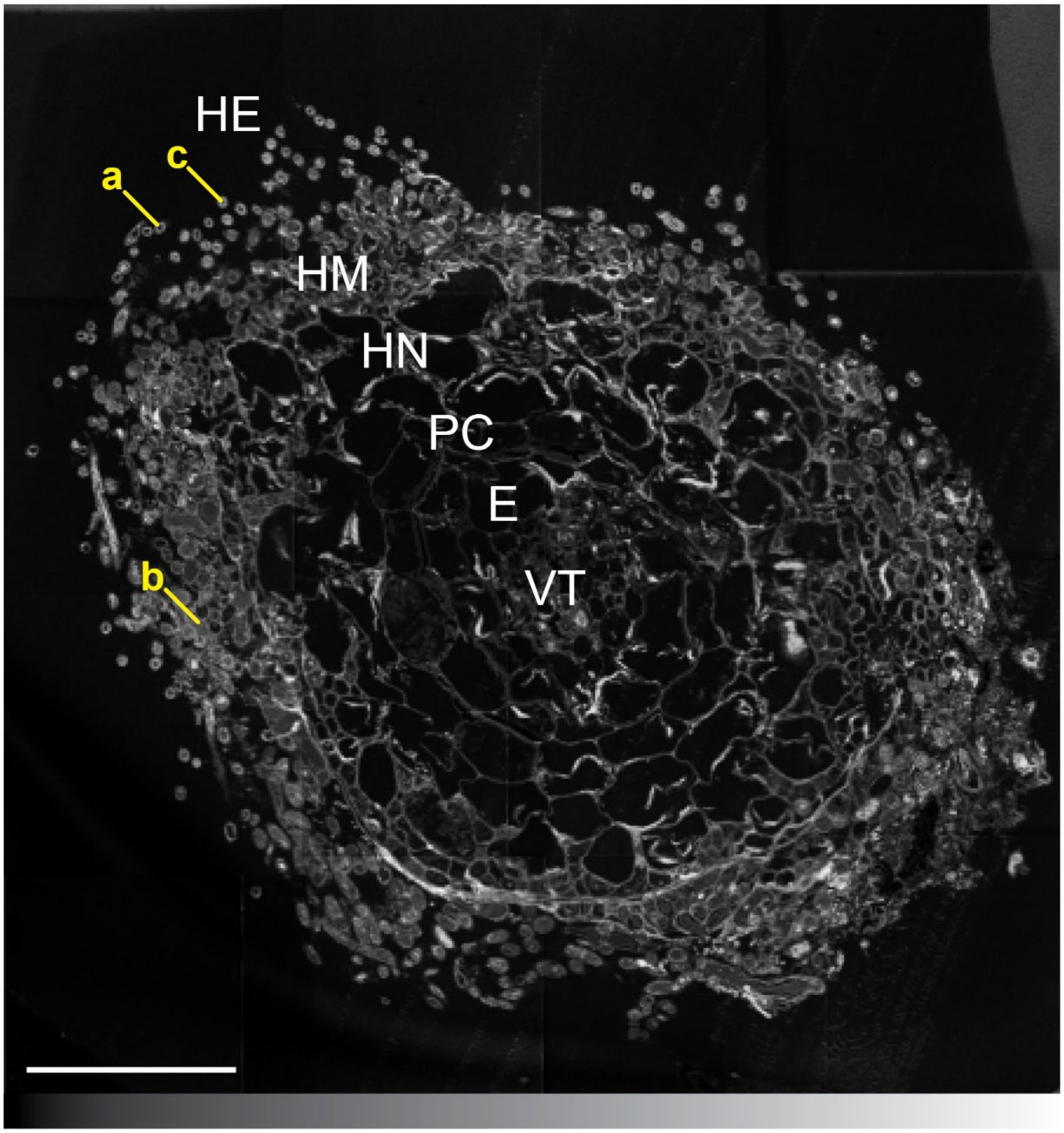

Table S1 Regression analysis of 13C vs. 15N isotope enrichment (at% excess, APE) as determined by NanoSIMS in distinct tissue types of an ectomycorrhizal root tip cross section of beech (Fagus sylvatica) and Thelephora fungi (Fig. S6 and S7, for a graphical representation of the correlations see Fig. 7) . Statistical model selection was based on the Akaike information criterion (AIC) and mean residual squared error (MSE): Linear (lm), single (ln 13C) and double ln transformed (13C and 15N, lnln) ordinary least squares regression (OLS) and segmented linear regression (breakpoint analysis, seg.lm). Histological tissue categories: HE – extended hyphae; HM – hyphal mantle; HN – hyphae Hartig net; plant: PC – plant cortex, E – endodermis. VT – vascular tissue; CW – cell wall and L – lumen

			**lm**		**lnln**		**ln ^{13}C**		**seg. lm**	
		**n**	**AIC**	**MSE**	**AIC**	**MSE**	**AIC**	**MSE**	**AIC**	**MSE**
**HE**	**CW**	425	1876.04	4.09E-32	735.12	1.73E-31	1892.35	5.37E-33	1680.52	3.93E-28
**HM**		371	1202.96	7.79E-35	457.83	6.38E-32	1158.18	4.77E-32	1154.99	4.60E-28
**HN**		322	1065.71	4.68E-34	341.25	1.32E-31	1059.05	1.58E-31	1056.04	1.90E-28
**PC**		82	158.38	1.35E-34	48.96	4.65E-33	147.91	4.94E-33	130.18	1.34E-30
**E**		34	86.13	3.00E-33	61.89	5.89E-32	87.13	1.97E-32	89.10	1.08E-29
**VT**		88	186.87	8.49E-31	100.68	7.25E-32	181.65	1.96E-31	184.83	4.03E-30
**HE**	**L**	217	1041.30	1.32E-32	506.25	7.70E-32	906.57	6.68E-34	968.33	3.44E-28
**HM**		225	1023.44	4.79E-34	418.39	4.06E-33	915.35	8.54E-34	948.39	5.21E-28
**HN**		124	543.97	6.61E-33	230.56	2.43E-36	498.40	7.15E-32	525.54	1.98E-28
**PC**		116	57.51	9.71E-36	224.91	2.05E-34	124.42	1.93E-33	17.66	1.12E-32
**E**		24	28.45	7.53E-36	38.44	8.43E-34	23.88	4.36E-34	19.84	2.39E-32
**VT**		61	136.82	4.02E-34	131.97	1.71E-33	121.92	7.71E-36	116.15	7.04E-31

Methods S1 Additional methodological details on NanoSIMS analysis

In order to prevent coeval detection of isobars (e.g $^{13}C^{14}N^-$ ions in addition to the targeted $^{12}C^{15}N^-$ ions), the mass spectrometer was tuned for achieving a mass resolving power (MRP) of > 10.000 (according to Cameca's definition). Secondary ion beam drift was corrected by automatic beam centering - utilizing the $^{12}C_2^-$ signal as reference - as well as automatic peak centering for each of the recorded secondary ion species after movement of the sample stage to the area of the following image acquisition.

Prior to data acquisition, the total analysis area was pre-sputtered utilizing a defocused Cs^+ ion beam (ca. 2,5 µm beam diameter) with 250 pA beam current in order to remove the AuPd coating and establishment of the secondary ion signal intensity steady-state regime. Due to the large size of the analysis area, pre-sputtering was performed consecutively over 4 individual scanning areas of (125 x 125 µm each) with 20 µm overlap at the inner edges of the squares. The primary ion fluence achieved within each of the 4 scanning areas was 7,5E16 ions/cm^2.

With respect to the measured ^{13}C contents, it should be noted that resin embedding introduces significant amounts of carbon with natural isotopic abundance into the sample resulting in severe attenuation of the relative ^{13}C enrichment (Kopf *et. al.*, 2015). In addition, selective elution of labelled compounds through resin embedding can lead to biasing of the detected isotopic compositions. As such, conclusions on the absolute amounts of the transferred nutrients are limited and need to be done carefully.

Signal intensities were corrected for detector dead-time and quasi-simultaneous arrival (QSA) of secondary ions (Slodzian *et al.*, 2001, 2004), utilizing QSA sensitivity factors ('beta-values') of 1.1, 1.06 and 1.05 for C^-, C_2^- and CN^- secondary ions, respectively. The determination of the beta values was conducted on cellular biomass (dried yeast cells, data not shown).

Notes S1 Potential bias of 15N measurements due to N2 adsorption during consecutive NanoSIMS analysis of multiple fields of view on one sample

We note that we performed two analysis runs. In the first run, image acquisition was conducted without additional pre-sputtering, which resulted in biasing of the ^{15}N isotope content within sample areas that were exposed to primary ion bombardment in acquisition of a preceding image (Fig. S2, S3). For the second run, we decreased the base pressure in the analysis chamber from 6E-10 mbar to 1,3E-10 mbar by titanium sublimation pumping (TSP) and implemented a 295 sec high intensity primary ion irradiation (100 pA) right before acquisition of each individual image stack. With these modifications, the deviation in the measured ^{15}N content no longer occurred and we conclude that the biasing observed in the first run originated from adsorption of nitrogen containing molecules (mainly N_2) with natural isotopic abundance in the residual gas. All data presented in this publication were acquired in the second analysis run.

References

Slodzian G, Chaintreau M, Dennebouy R, Rousse A. **2001**. Precise in situ measurements of isotopic abundances with pulse counting of sputtered ions. *The European Physical Journal Applied Physics* **14**: 199–231.

Slodzian G, Hillion F, Stadermann FJ, Zinner E. **2004**. QSA influences on isotopic ratio measurements. *Applied Surface Science* **231**–**232**: 874–877.

Kopf S. H., McGlynn S. E., GreenSaxena A., Guan Y., Newman D. K., Orphan V. J. 2015. Heavy water and ^{15}N labelling with NanoSIMS analysis reveals growth rate - dependent metabolic heterogeneity in chemostats. *Environmental microbiology* **17(7)**: 2542-2556
